# BRAHMA ATPase of the SWI/SNF Chromatin Remodeling Complex Acts as a Positive Regulator of Gibberellin-Mediated Responses in Arabidopsis

**DOI:** 10.1371/journal.pone.0058588

**Published:** 2013-03-11

**Authors:** Rafal Archacki, Daniel Buszewicz, Tomasz J. Sarnowski, Elzbieta Sarnowska, Anna T. Rolicka, Takayuki Tohge, Alisdair R. Fernie, Yusuke Jikumaru, Maciej Kotlinski, Roksana Iwanicka-Nowicka, Katarzyna Kalisiak, Jacek Patryn, Joanna Halibart-Puzio, Yuji Kamiya, Seth J. Davis, Marta K. Koblowska, Andrzej Jerzmanowski

**Affiliations:** 1 Department of Plant Molecular Biology, Faculty of Biology, University of Warsaw, Warsaw, Poland; 2 Institute of Biochemistry and Biophysics, Polish Academy of Sciences, Warsaw, Poland; 3 Max-Planck Institute for Plant Breeding, Cologne, Germany; 4 Max Planck Institute of Molecular Plant Physiology, Potsdam-Golm, Germany; 5 RIKEN Plant Science Center, Tsurumi-ku, Yokohama, Kanagawa, Japan; Instituto de Biología Molecular y Celular de Plantas, Spain

## Abstract

SWI/SNF chromatin remodeling complexes perform a pivotal function in the regulation of eukaryotic gene expression. Arabidopsis (*Arabidopsis thaliana*) mutants in major SWI/SNF subunits display embryo-lethal or dwarf phenotypes, indicating their critical role in molecular pathways controlling development and growth. As gibberellins (GA) are major positive regulators of plant growth, we wanted to establish whether there is a link between SWI/SNF and GA signaling in Arabidopsis. This study revealed that in *brm-1* plants, depleted in SWI/SNF BRAHMA (BRM) ATPase, a number of GA-related phenotypic traits are GA-sensitive and that the loss of BRM results in markedly decreased level of endogenous bioactive GA. Transcriptional profiling of *brm-1* and the GA biosynthesis mutant *ga1-3*, as well as the *ga1-3/brm-1* double mutant demonstrated that BRM affects the expression of a large set of GA-responsive genes including genes responsible for GA biosynthesis and signaling. Furthermore, we found that BRM acts as an activator and directly associates with promoters of *GA3ox1,* a GA biosynthetic gene, and *SCL3*, implicated in positive regulation of the GA pathway. Many GA-responsive gene expression alterations in the *brm-1* mutant are likely due to depleted levels of active GAs. However, the analysis of genetic interactions between BRM and the DELLA GA pathway repressors, revealed that BRM also acts on GA-responsive genes independently of its effect on GA level. Given the central position occupied by SWI/SNF complexes within regulatory networks controlling fundamental biological processes, the identification of diverse functional intersections of BRM with GA-dependent processes in this study suggests a role for SWI/SNF in facilitating crosstalk between GA-mediated regulation and other cellular pathways.

## Introduction

The SWI/SNF chromatin remodeling complexes are evolutionarily conserved multimeric assemblages of proteins that use the energy of ATP hydrolysis to disrupt DNA-histone interactions. Through their ability to regulate access to nucleosomal DNA they exert profound effects on transcriptional activity [1]. SWI/SNF-mediated chromatin remodeling has been shown to play a central role in cell proliferation, differentiation and development [2]. All SWI/SNF complexes possess a catalytic subunit (ATPase) associated with a set of accessory core subunits, including homologs of yeast SNF5 and SWI3 proteins which are essential for assembly, overall stoichiometry and recruitment of SWI/SNF to target loci [3], [4]. Arabidopsis has two major orthologs of the ATPase (BRM and SYD) and four orthologs of SWI3 (SWI3A, SWI3B, SWI3C, SWI3D), which gives the potential to assemble complexes with different combinations of subunits [5], [6]. As global regulators the Arabidopsis SWI/SNF complexes are essential. This is reflected by the embryo-lethal phenotypes of single *swi3a* and *swi3b* mutants and of double *brm/syd* mutants [7], [8]. Due to partial redundancy between the BRM and SYD ATPases, single mutants in their respective genes are viable. The phenotypes of these mutants, and of mutants in the SWI3C and SWI3D subunits, are dwarf or semi-dwarf with numerous aberrations in organ development [9], [10], [7].

While some of the processes disrupted in *swi/snf* mutants have been revealed [11], [12], [13], the global pattern of changes in the regulatory networks that could lead to their strong and complex developmental phenotypes is largely unknown. Recently, SYD and BRM ATPases were shown to interact with LEAFY and SEPALLATA3 proteins in order to control floral organ identity, acting antagonistically to Polycomb repressors [14]. There is also evidence linking SWI/SNF complexes with hormonal pathways. SYD is involved in the regulation of jasmonic acid- and ethylene-dependent genes [15], and SWI3B is an interaction partner of HAB1, a key element in ABA signaling [16]. A transcriptional profiling study of *brm* and *syd* null mutants identified that several genes involved in auxin and GA signaling were affected [8]. These data and the properties of Arabidopsis *swi/snf* mutants prompted us to examine whether there is a functional link between GA signaling and SWI/SNF complex-mediated chromatin remodeling.

GAs are major promoters of plant growth and development that are involved in various processes including seed germination, vegetative growth, flowering and stress responses [17], [18], [19]. Levels of active GAs are tightly controlled through transcriptional regulation of genes encoding GA 20-oxidases (GA20ox) and GA 3-oxidases (GA3ox), responsible for the late steps of GA biosynthesis, as well as GA 2-oxidases (GA2ox), responsible for GA degradation [20], [21], [22]. GA signaling initiates with the binding of GA to one of its receptors (GID1a, b, and c in Arabidopsis), triggering proteasomal degradation of the master growth repressors: the DELLA proteins [18], [19], [23], [24], [25]. Arabidopsis has five DELLA proteins: RGA, GAI and RGL1-3. Genetic analyses have shown that the different DELLAs perform both specific and overlapping functions during development [26], [27]. At low GA concentrations, DELLA proteins accumulate and act as repressors of growth and other GA-regulated developmental processes [28], [29], [30]. Gain-of-function *DELLA* mutants, or mutants with decreased levels of active GA, like *ga1-3*, which is defective in an early-step of GA biosynthesis, are characterized by a dwarf phenotype and strongly impaired germination, flowering, and fertility. Conversely, loss-of-function *DELLA* mutations lead to suppression of the *ga1-3* phenotype [27], [31]. More recently, DELLAs were found to interact with the light-responsive transcription factors PIF3 and PIF4 in the nucleus, prompting a model in which they act primarily by inhibiting transcriptional regulators [32], [33], [34], [35], [36], [37], [38], [39]. In addition to proteasome-dependent regulation of DELLA levels by active GA, their activities are also controlled by other proteins such as the N-acetylglucosamine transferase SPINDLY [40], [41], and SCARECROW-LIKE 3 (SCL3) that was recently proposed to act as an attenuator of DELLAs [42], [43].

In this study, we demonstrate that BRM, a catalytic subunit of SWI/SNF complexes, affects the expression of a significant number of GA-responsive genes in an opposite manner to DELLAs. This is consistent with our finding that the level of active GA is markedly decreased in the *brm* null mutant. Moreover, we show that BRM activates *GA3ox1* and binds to chromatin in the vicinity of its promoter, suggesting that it plays a direct role in the positive regulation of GA biosynthesis. However, we also show by genetic analyses, that BRM controls a number of GA-responsive genes independently of its effect on GA biosynthesis. We also reveal that in addition to targeting *GA3ox1,* BRM positively regulates and directly associates with the promoter of *SCL3*, a gene performing regulatory functions in the GA pathway. Our demonstration that BRM interacts with the GA signaling pathway at different levels, is the first evidence for participation of SWI/SNF chromatin remodeling in the mediation of GA responses.

## Results

### Plants Depleted of BRM Show GA-related Phenotypic Traits and Increased Sensitivity to GA Biosynthesis Inhibitor

**Figure 1:**
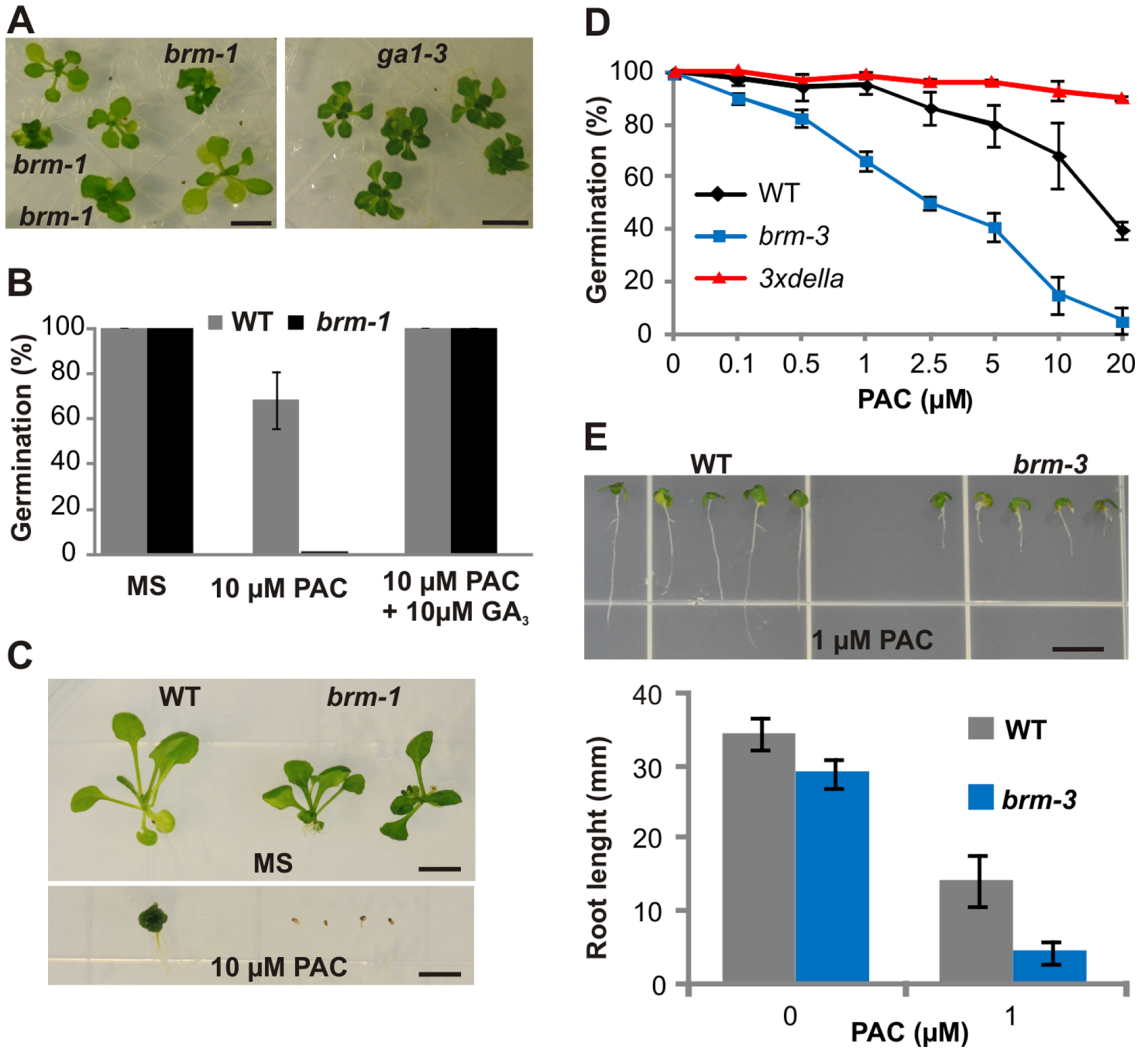
*brm* mutants show GA-related phenotypic traits and increased sensitivity to paclobutrazol. (A), Comparison of *brm-1* and *ga1-3* mutants grown on ½ MS medium for 18 days under long-day conditions. (B), Germination of the *brm-1* mutant is abolished in the presence of 10 µM PAC and rescued upon addition of exogenous gibberellin. The progeny of *brm-1/BRM* plants were analyzed 14 days after sowing. (C), Phenotype of *brm-1* plants grown for 25 days on 10 µM PAC after incubation of seeds with exogenous GA. (D), Germination assay of wild type, *brm-3* and *3xdella* (*rga/rgl1/rgl2*) lines. Seed coat rupture after 14 days was scored as germination. (E), Root elongation assay of wild type and *brm-3* plants grown for 12 days on PAC-containing medium. Bars in A, C and E = 5 mm.

Arabidopsis *brm* null mutants (such as *brm-1* and its phenocopy *brm-6*, studied here) [9], [44], are depleted in the SWI/SNF-type ATPase BRM and display a dwarf phenotype with characteristic short and branched roots, dark green coloration, closed flowers, underdeveloped stamens, male sterility and delayed flowering under non-inductive short-day conditions. In these respects, they resemble to some extent the phenotype of mutants with suppressed GA signaling or biosynthesis, such as *ga1-3* [45] or *ga3ox* [20], which have reduced levels of endogenous gibberellins (Figure 1A, Figure S1A–E). However, the *brm* null plants also display a number of unique features, like curled leaves and homeotic changes in flowers [9], [44], which are not shared by GA biosynthesis mutants. The GA-related phenotypic traits of *brm* null mutant are consistent with the microarray transcript profiling of Bezhani et al. (2007) [8] who reported that several hormone pathway genes, including those of the GA-pathway, are mis-expressed in *brm-101* (another *brm* null allele) and *syd-2* mutants.

The above data suggest that BRM plays a role in GA biosynthesis and/or signaling. To test this hypothesis, we examined *brm-1* phenotypes in the presence of the GA biosynthesis inhibitor, paclobutrazol (PAC). The *brm-1* plants were more sensitive to PAC than wild type plants, since *brm-1* homozygotes could not be recovered after germinating *brm-1/BRM* progeny on medium containing 10 µM PAC, the concentration at which wild type plants displayed a germination rate of about 70% (Figure 1B). We also tested growth responses of *brm-1* plants grown from seeds incubated with exogenous GA to ensure germination. The presence of 10 µM PAC severely affected the development of *brm-1* mutants, which failed to survive beyond 25 days, while wild type plants continued to grow under these conditions (Figure 1C). As PAC can possibly interfere with the biosynthesis of other hormones, we also examined growth on medium containing 10 µM PAC supplemented with exogenous GAs. In this case, the *brm-1* plants germinated and were viable, suggesting that the enhanced reaction of these mutants to PAC is linked with its inhibitory effects on the GA pathway (Figure 1B and Figure S1F).

Since *brm-1* is a highly pleiotropic mutant, defects in many different functions could have potentially influenced the outcome of the PAC assays and the PAC hypersensitivity of *brm-1* plants might be due to the additive effect of GA deficiency and earlier defects in growth and/or development resulting in the dwarf stature of adult *brm-1* plants. To examine this possibility, we used a weak *brm-3* mutant in which a T-DNA insertion in the 3′ portion of the *BRM* gene gives rise to a truncated protein lacking a C-terminal fragment of 454 amino acids (approximately 1/5th of the molecule). Although the *brm-3* mutant exhibits only mild developmental and growth defects (Figure S2) [46], PAC treatment had a much greater inhibitory effect on the germination of *brm-3* than on wild type seeds, while germination on medium without PAC was normal for both the *brm-3* and the wild type. As expected, a *triple della* mutant (*rga/rgl1/rgl2*) was insensitive to PAC treatment (Figure 1D). Moreover, in the presence of PAC, *brm-3* had significantly shorter roots than wild type plants (Figure 1E). Thus, both null and weak *brm* mutations confer increased sensitivity to a GA biosynthesis inhibitor, indicating that this phenotypic trait is not a secondary effect of earlier GA-independent growth defects caused by the *brm* mutation.

### *brm* Null Mutants Show Both GA-sensitive and -insensitive Traits and have Reduced Levels of Bioactive GA

**Figure 2:**
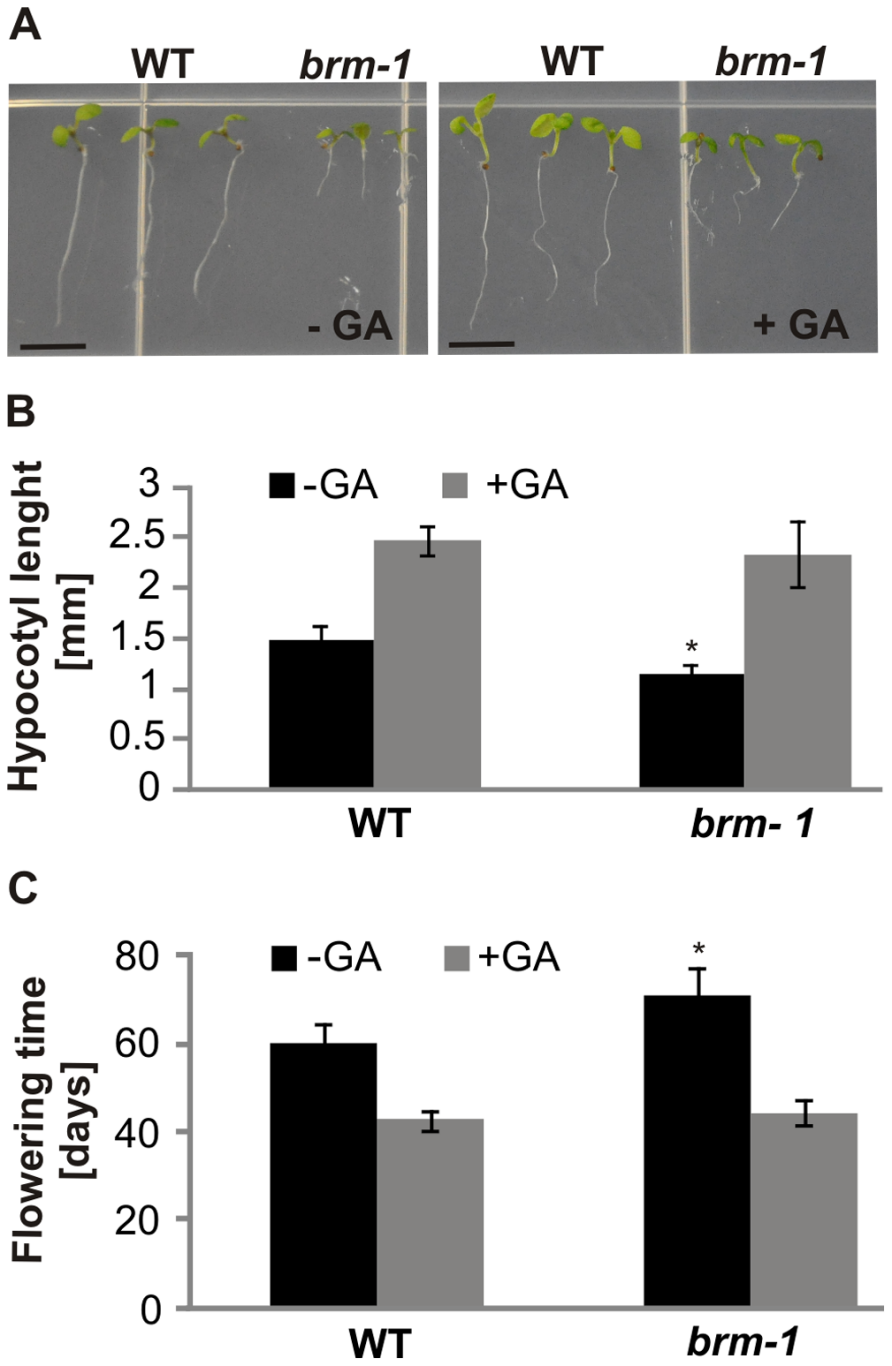
GA responses of the *brm-1* mutant. (A, B), Elongation of *brm-1* hypocotyls and roots in response to 1 µM GA_4_. Plants were grown on ½ MS medium for 8 days under long-days conditions in the presence or absence of 1 µM GA_4_. GA application caused considerable elongation of the hypocotyls, but had little effect on *brm-1* root growth. Bar = 5 mm. (B), Hypocotyl length of plants grown as in A. Presented data are the means of 12 measurements ± s.d. (C), Flowering of *brm-1* plants in response to exogenous gibberellins. Plants were grown in soil under short-day conditions and treated with 10 µM GA_3_. At least 15 plants of each line/condition were scored. Data are the means ± s.d. Asterisks indicate significant differences from the wild type plants (p<0.01).

To learn more about the relationship between GA signaling and BRM-mediated processes, we examined the responsiveness of the *brm-1* mutant to exogenous GAs. The ability to germinate on PAC+GA medium (Figure 1B) indicated that the *brm* null mutation does not abolish GA perception. Moreover, in the presence of GA, *brm-1* plants displayed significantly increased hypocotyl growth (Figure 2A, B) and a greatly accelerated onset of flowering under short-day conditions (i.e. days to flowering), such that the mutants flowered similarly to wild type plants (Figure 2C, Table S1). This suggested that at least some traits of *brm* mutants may be caused by GA deficiency, which can thus be overcome by application of exogenous GAs, as is the case for GA biosynthesis mutants.

Therefore, we next compared, through combined liquid chromatography-mass spectrometry [47], the levels of key metabolites in the GA biosynthesis and degradation pathways, and of GA_4_, a predominant bioactive form of GA in Arabidopsis [48], [22], in 4-week old wild type, *brm-6* (*brm* null allele) [44] and *ga1-3* (in Col-0 ecotype) [27] plants grown in soil. Levels of GA_4_ in *brm-6* and *ga1-3* plants were around 50% and 15% of the wild type value, respectively (Table 1). Analysis of GA_34_, an inactive catabolite derived from active GA_4_, showed that its level was strongly reduced in *brm-6* plants, indicating that the decrease in active GA_4_ in this mutant resulted from defective GA biosynthesis rather than enhanced GA degradation. Interestingly, the level of GA_12_, but not GA_9_ (a direct precursor of GA_4_), was also reduced in *brm-6* compared with the wild type (Table 1). The relatively high level of GA_9_ is probably due to the decreased rate of its conversion to GA_4_, while the reduced level of GA_12_ might be caused by increased 20-oxidation of this form (see Discussion).

**Table 1:** Concentration of gibberellins in wild type and mutant lines.

	GA_12_	GA_9_	GA_4_	GA_34_
wild type	5.206 (0.327)	1.730 (0.105)	4.269 (0.315)	6.153 (0.159)
*brm-6*	2.782 (0.258)	2.063 (0.384)	1.911 (0.186)	2.697 (0.125)
*ga1-3*	n.d.	n.d.	0.583 (0.072)	0.095 (0.095)

The values are ng/g dry weight (s.e.). They are the means of three biological replicates except for the GA_12_ and GA_9_ measurements in *ga1-3*, for which 2 replicates were used. n.d. – not determined.

The above analysis indicated that the *brm* null mutant is partly GA deficient, suggesting a direct or indirect role of BRM in GA biosynthesis. However, unlike in typical GA-deficient mutants, treatment with GA did not reverse some of the other *ga1-3*-like traits of *brm-1* plants. Although the cotyledon size, rosette radius and stem height of *brm-1* mutant plants increased upon GA treatment, they remained much smaller than in wild type plants (Figure 2A, Table S1). In addition, when grown in the presence of GA, the roots of *brm-1* remained significantly shortened compared with those of wild type plants (Figure 2A). There was also no reversion by GA treatment of *brm-1* flower phenotypes, and the mutant plants remained completely male sterile (not shown). Thus, a number of the GA deficiency-like aberrant traits caused by the *brm* null mutation (short stature, short roots, flower defects and male sterility) did not change or changed only slightly upon GA treatment. Some of these *brm* mutant traits might be strongly influenced by defects in processes that involve BRM, but are independent of the GA pathway, while others could be caused by GA-insensitive defects in GA signaling.

### BRM Affects the Expression of Both GA Biosynthesis and Signaling Genes

**Figure 3:**
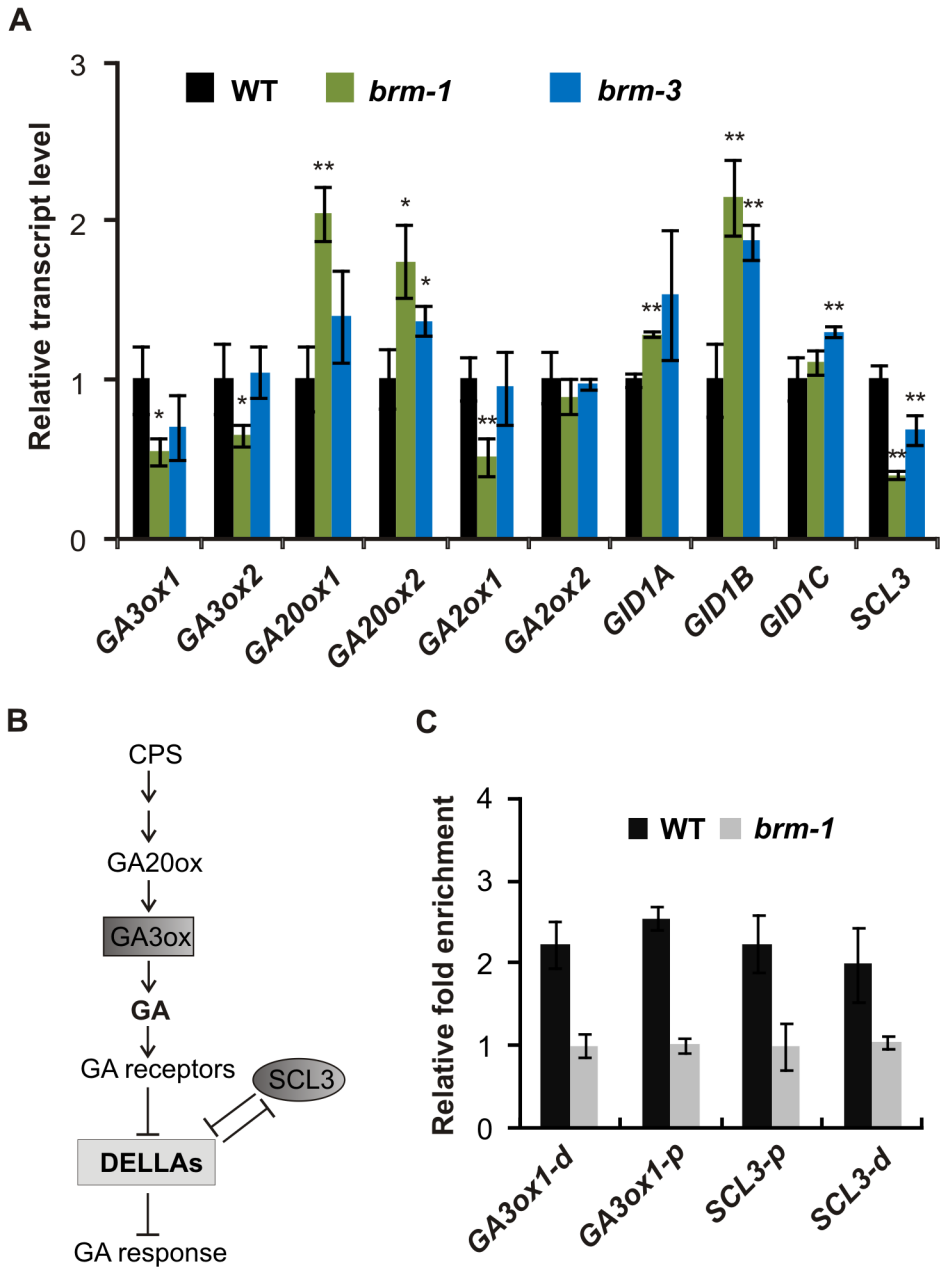
BRM directly regulates the expression of the *GA3ox1* and *SCL3* genes. (A), RT-qPCR analysis of relative transcript levels of GA biosynthesis and signaling genes in 18-d-old wild type, *brm-1* and *brm-3* lines. The housekeeping genes *PP2A* and *GAPC* were used as normalization controls. RT-qPCR data are the means ± s.d. of 3 biological replicates. Transcript levels in the wild type were set to 1. Asterisks indicate significant differences from the wild type plants with p<0.05 (*) or p<0.01 (**). (B), Simplified model of the GA signaling pathway. (C), BRM recruitment to the promoters of *GA3ox1* and *SCL3* in wild type and *brm-1* plants, analyzed by ChIP-qPCR. The signal obtained for the *PP2A* promoter region was used to normalize the qPCR results in each sample. Distal (d) and proximal (p) promoter sequences relative to the start codon of each gene were analyzed. Fold enrichment of each region in the wild type was calculated relative to the *brm-1* sample. The value of ChIP enrichment in *brm-1* was set to 1. Data are the means ± s.e. from 3 reactions in one ChIP experiment. Similar results were obtained in separate experiments.

Since the above results implicated BRM in GA biosynthesis and also suggested a role in GA signaling, we used qRT-PCR to examine the expression of genes encoding enzymes involved in the late steps of GA biosynthesis (*GA20ox1*, *GA20ox2*, *GA3ox1*, *GA3ox2*) and GA inactivation (*GA2ox1*, *GA2ox2*), as well as genes coding for GA receptors (*GID1a*,b,c) and *SCARECROW-LIKE 3* (*SCL3*), which is believed to act in the GA pathway by attenuating the DELLA repressors [42], [43]. The levels of the *GA3ox1, GA3ox2*, *GA2ox1*, and *SCL3* transcripts were significantly decreased in *brm-1* compared with wild type plants. The abundance of the *GA20ox1* and *2, GID1a* and *b* transcripts was increased, while *GA2ox2* and *GID1c* were not significantly changed compared with the wild type (Figure 3A). To confirm these data, we then examined levels of the same transcripts in the weak *brm-3* mutant. Consistent with its increased sensitivity to PAC (Fig. 1D and E), the expression of the majority of GA pathway genes (except *GA3ox2* and *GA2ox1*) was also changed, albeit slightly, in this mutant (Figure 3A). The relatively small changes in expression in *brm-3* correspond to the mild phenotypic effects seen in this mutant under normal conditions, and are similar to the findings of a previous study showing less severe changes in the expression of homeotic genes in *brm-3* than in *brm-1* [46]. The results of these analyses confirmed that GA pathway genes are mis-regulated in null and weak *brm* mutants.

### BRM Occupies the Promoters of the *GA3ox1* and *SCL3* Genes

The decreased expression of *GA3ox1* is likely to be the primary cause of the reduced GA_4_ content in *brm* null mutants, since the GA3ox1 enzyme catalyzes conversion of precursor GA_9_ to GA_4_ in the final step of GA biosynthesis in Arabidopsis [22]. GA biosynthesis and GID1 genes are known to be feedback regulated by GAs. The *GA20ox* and *GID1* genes are up-regulated, whereas *GA2ox* genes are downregulated under low GA conditions [23], [49], [50]. We thus hypothesized that increased expression of *GA20ox* and *GID1* genes, as well as decreased expression of the *GA2ox1* gene in *brm* mutants is a secondary effect caused by a feedback mechanism compensating downregulation of *GA3ox1* and depletion of active GA, and possibly also downregulation of *SCL3*, which encodes an important factor involved in the maintenance of GA pathway homeostasis (Figure 3B) [25]. Therefore, we examined whether BRM regulates *GA3ox1* and *SCL3* expression by directly interacting with their regulatory sequences. A chromatin immunoprecipitation (ChIP) assay was performed using wild type and *brm-1* plants and anti-BRM antibody [44]. Enrichment of *GA3ox1* and *SCL3* promoter sequences was detected, while there was no significant enrichment of promoter sequences of *GA20ox2* or *GID1b* (Figure 3C, Figure S3). Thus, the *GA3ox1* and *SCL3* genes represent direct targets of BRM, which is consistent with the notion that BRM is involved in the regulation of both GA biosynthesis and GA signaling.

### *ga1-3/brm-1* Double Mutant Shows Additive and Synergistic Traits

**Figure 4:**
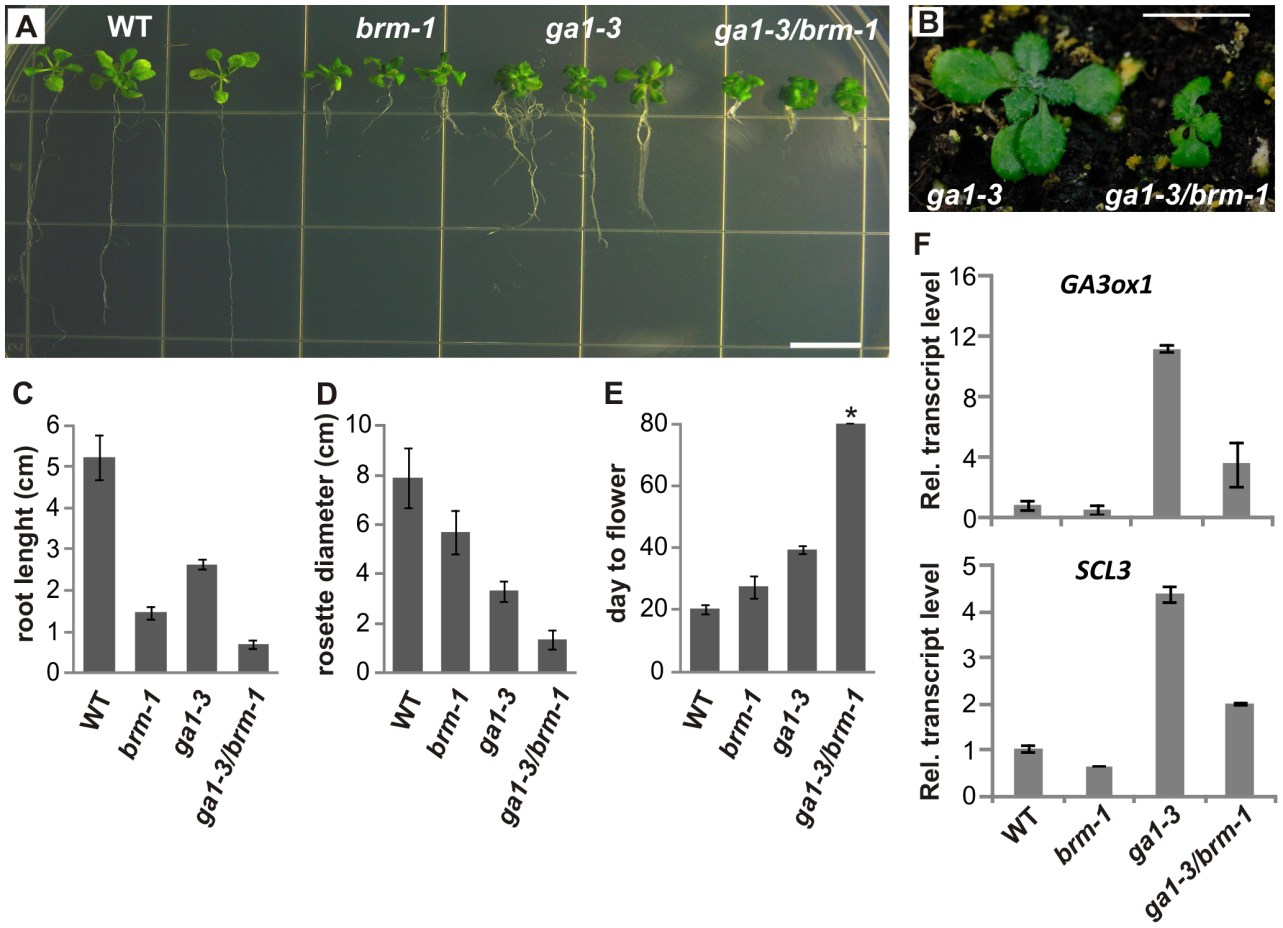
*ga1-3/brm-1* mutant phenotypes. (A–B), Phenotypes of the *ga1-3*, *brm-1* and *ga1-3/brm-1* mutants grown on MS medium (18-d-old seedlings, A) or in soil (22-d-old plants, B). Bars = 10 mm. (C–F), Quantitative characterization of *brm-1*, *ga1-3* and *ga1-3/brm-1* mutants: root length of 18-d-old seedlings (C), rosette diameter at maturity (D) and flowering time under LD conditions (E). Data are the means ± s.d., 10 plants of each line were scored, except for *ga1-3/brm-1* (7 plants). * All *ga1-3/brm-1* plants except one failed to flower by the end of the experiment (80 days). (F), RT-qPCR analysis of relative transcript levels of *GA3ox1* and *SCL3* in 20-d-old wild type, *brm-1*, *ga1-3*, and *ga1-3/brm-1* lines. RT-qPCR data are the means ± s.d. of 3 biological replicates. Transcript levels in the wild type were set to 1.

In order to genetically test the contribution of BRM to GA-related responses, we generated a *ga1-3/brm-1* double mutant line. When compared with either of the single mutants, homozygous *ga1-3/brm-1* plants showed a number of additive as well as synergistic traits including increased dwarfism, very short roots and the inability to flower under long-day conditions (Figure 4A–E; Figure S4). Double mutant plants were also less viable when grown in soil: only a few *ga1-3/brm-1* homozygotes were recovered after germinating about 1000 heterozygous *ga1-3/brm-1/BRM* seeds. This could be due to the severely underdeveloped roots of *ga1-3/brm-1* (Figure 4A, C). As the GA level in *ga1-3* is at least 3-fold lower than in *brm-1*, the additive traits of the double *brm-1/ga1-3* mutant could be the result of GA pathway-independent functions of BRM. However, enhancement of the *ga1-3* phenotype has also been reported for mutants in genes acting as positive regulators of downstream GA responses, such as *scl3* [42], [43]. Therefore, the observed additive effect could also be due, at least in part, to down-regulation of *SCL3*. Nonetheless, in spite of the enhanced phenotype of the *ga1-3/brm-1* double mutant, the growth of *brm-1* plants on 10 µM PAC (Figure 1B) resulted in a stronger phenotype. This could be explained by the low amount of GAs present in the *ga1-3* mutant ([51], this work) and the possible blocking effect of PAC on other signaling pathways. The *ga1-3* phenotype was shown to be further strengthened by PAC treatment [52], which is consistent with both of these explanations.

We next examined the levels of *GA3ox1* and *SCL3* transcripts in the *ga1-3/brm-1* double mutant in comparison with the *ga1-3* single mutant. The transcripts of *GA3ox1* and *SCL3* are known to be up-regulated under low GA conditions as part of the feedback regulation of the GA pathway [23], [25]. RT-qPCR analysis demonstrated that the *brm* null mutation causes a marked decrease in *GA3ox1* and *SCL3* transcript levels in the *ga1-3* background (Figure 4F), indicating that BRM also contributes to the regulation of these genes under low GA conditions. The overall effects of the double *ga1-3/brm-1* mutation suggest that BRM, in addition to positively regulating the GA level, probably functions as a regulator of GA responses, at least in part by promoting *SCL3* expression.

### The Transcriptional Profile of *brm-1* Overlaps with that of the *ga1-3* Mutant

**Figure 5:**
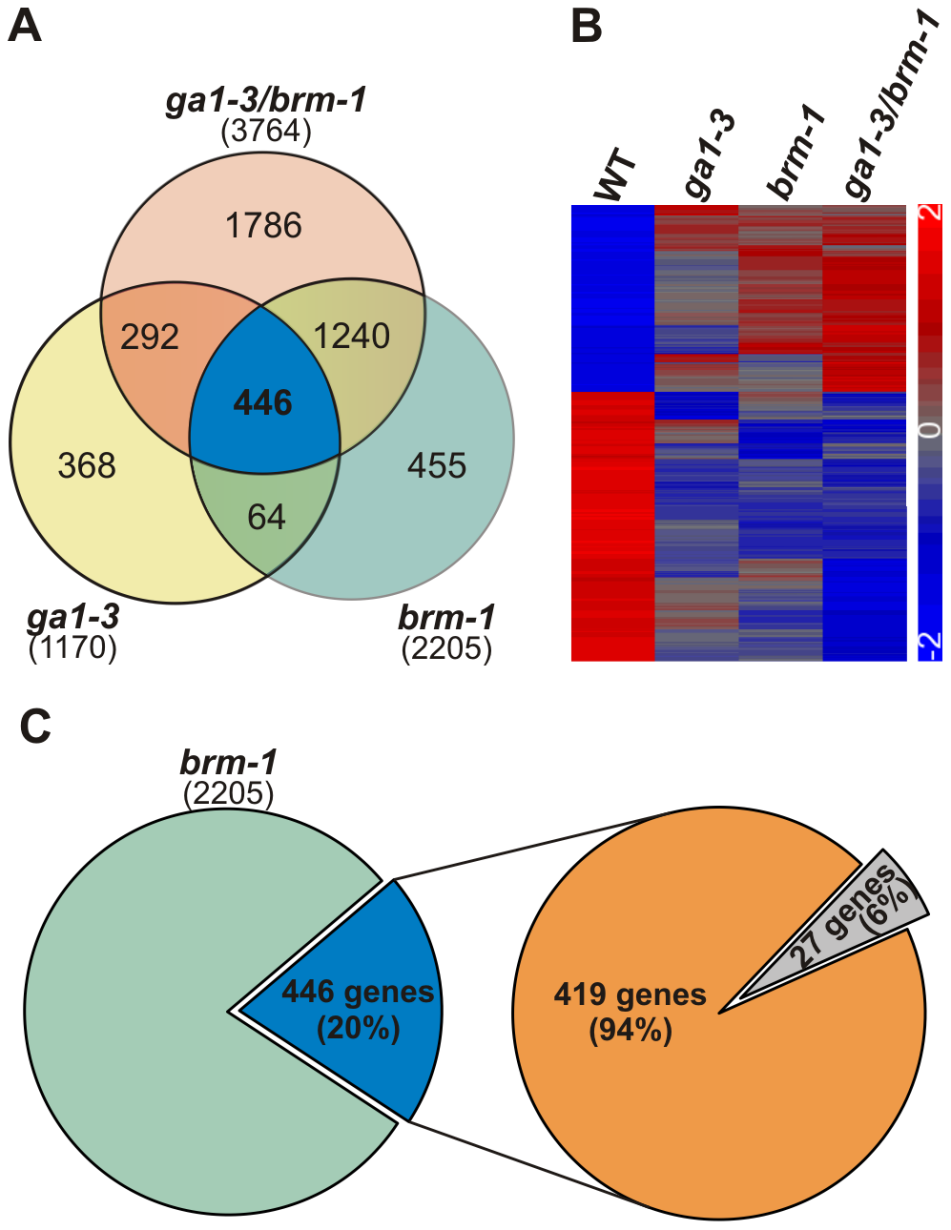
Transcriptional profile of *brm-1* overlaps with that of *ga1-3*. (A), Overlap between differentially regulated genes in mutants *brm-1*, *ga1-3* and *ga1-3/brm-1* identified in microarray data, shown by a Venn diagram. (B), Genes up- and down-regulated in all three mutants, shown by a heat map. The color scale represents normalized expression levels. (C), 94% of the genes commonly mis-expressed in all three mutants show expression changes in a similar direction. Green – genes mis-regulated only in *brm-1*; blue – genes mis-regulated in *brm-1*, *ga1-3* and *ga1-3/brm-1*; orange – genes mis-regulated in a similar direction in all three mutants; gray – genes mis-regulated in an opposite direction in one of the mutants.

To investigate how BRM contributes to global GA-dependent transcriptional regulation, we compared the transcript profiles of 18-d-old seedlings of *ga1-3*, *brm-1* and double *ga1-3/brm-1* mutant lines and wild type plants, grown on MS medium (see Figure 4A; Tables S2, S3, S4). Microarray transcriptome analysis revealed a considerable overlap between the single *brm-1* and *ga1-3* mutants: over 40% of genes that were mis-expressed in *ga1-3* (compared with the wild type), also showed mis-expression in *brm-1* (Figure 5A, B; Table S5). This showed that the expression of a significant number of GA-responsive genes is also dependent on the BRM-containing SWI/SNF complex, which is consistent with a positive role for BRM in GA biosynthesis and signaling. In agreement with our qRT-PCR data, the two direct targets of BRM, *GA3ox1* and *SCL3,* were present among the genes down-regulated in *brm-1* compared with the wild type (among recognized GA biosynthesis and signaling genes, the microarray and qRT-PCR data were inconsistent only for *GA20ox2*; Tables S2–S3).

Functional classification based on Gene Ontology showed that overall, the *brm-1* mutation had a much broader effect on gene expression than *ga1-3* (Figure S5A, B), confirming that BRM regulates many processes independent of gibberellins. However, gene clusters commonly regulated by both BRM and GAs were also identified (Table 2; Figure S5). In both the *ga1-3* and *brm-1* gene sets, genes involved in stress responses, the circadian clock, flowering, and responses to light and hormones were highly enriched (Table 2). These processes are known to be influenced by gibberellins [53], [54], [55], [56], [32] and were also enriched in transcriptional analyses of DELLA-responsive genes [57], [58], [59]. Interestingly, in our analysis, the greatest enrichment in both mutant gene sets was in genes involved in responses to auxin stimulus which is consistent with the extensive cross-talk between the GA and auxin pathways [59], [60], [61] (Table 2, Figure S5A). Moreover, examination of the molecular function categories identified a highly enriched cluster of genes encoding carboxylesterases and pectin-related enzymes, that was similarly affected in *ga1-3* and *brm-1* mutants (Figure S5B, C), indicating that BRM can regulate large GA-dependent gene families.

**Table 2:** Gene Ontology (GO) categories statistically over-represented among genes differentially expressed in both *ga1-3* and *brm-1* mutants.

GO category (biological process)	p-value	Number of genes
response to auxin stimulus	3.89E−10	29
circadian rhythm	1.90E−4	8
response to red or far red light	6.55E−4	13
cellular carbohydrate metabolic process	0.0169	21
photoperiodism, flowering	0.0360	5
response to gibberellin stimulus	0.0445	8
response to salt stress	0.0461	15

The inclusion of data for the double *ga1-3/brm-1* mutant revealed a common set of 446 genes mis-expressed in all three mutants (Figure 5A; Table S6). It is noteworthy that over 90% of genes in this overlapping group displayed a similar pattern of mis-expression (either up- or down-regulation) in the *brm-1* and *ga1-3* mutant backgrounds (Figure 5C). This showed that BRM depletion (*brm-1* mutant) or a significant decrease in GA content (*ga1-3* mutant) have a similar effect on gene expression, further supporting a positive role for BRM in GA biosynthesis. Consistent with this finding, the majority of these genes (about 60%) showed non-additive expression levels in the double mutant compared with the single mutants. It is likely that these genes react mainly to decreased GA levels caused by both *brm-1* and *ga1-3* mutations. Interestingly, the remaining genes from the overlapping group (about 40%) exhibited enhanced mis-expression in the double mutant compared with both single mutants (Figure 5B; Table S7), consistent with the more severe phenotypic effects observed in *ga1-3/brm-1* plants. The changes in gene expression in this sub-group were mostly additive, although for some of the analyzed genes, they could be classified as synergistic, as the *ga1-3* and *brm-1* mutations enhanced the action of one another (Table S7). The additive changes, as hypothesized above with respect to phenotypic changes, could be due to GA-independent effects of the *brm-1* mutation on GA-responsive genes. One possible explanation for the genes affected synergistically, is that GA signaling and BRM-mediated chromatin remodeling converge on the same targets with some functional interactions.

### DELLA Mutations Partially Suppress the *brm* Phenotype

**Figure 6:**
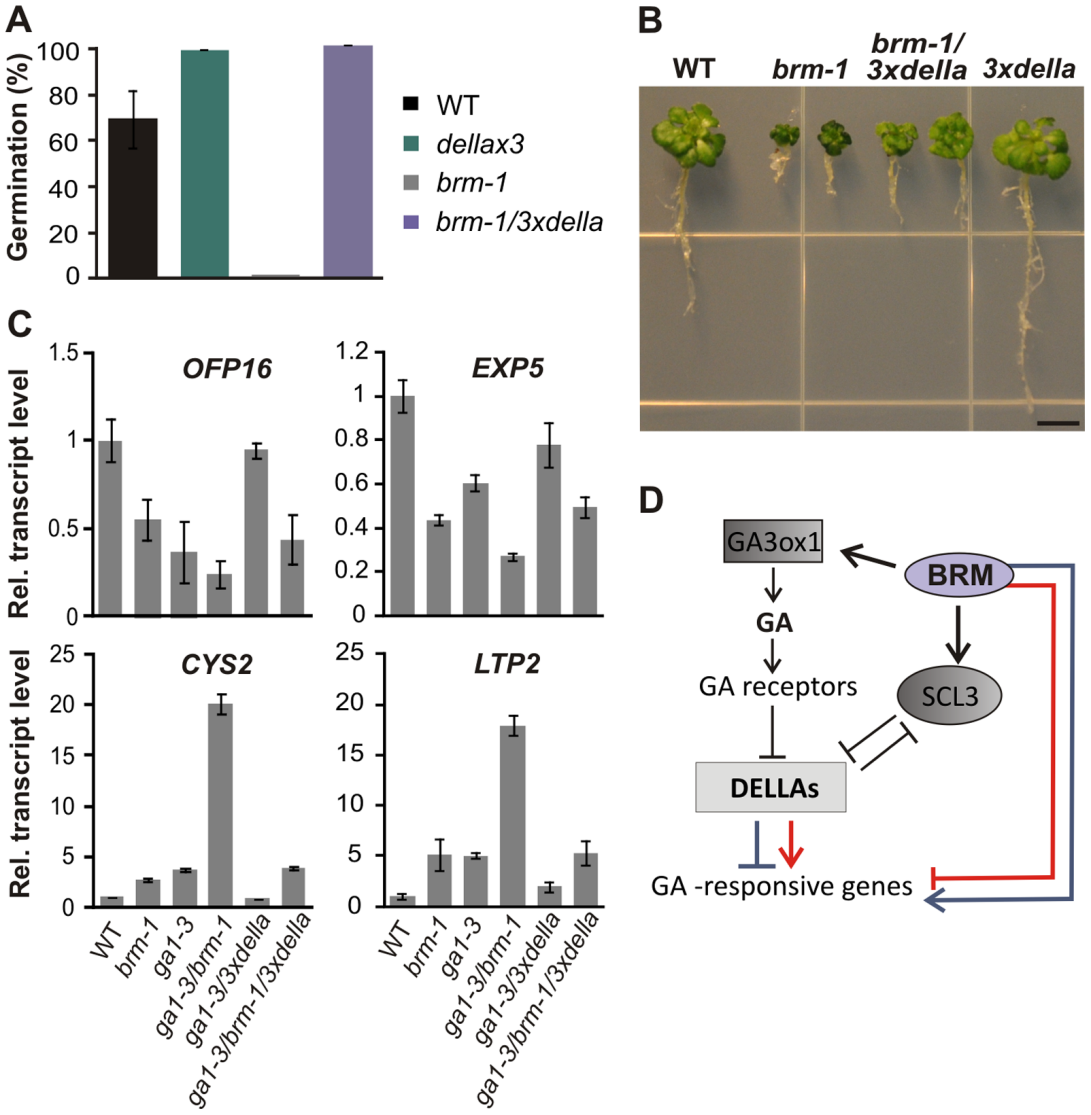
BRM acts through distinct mechanisms to regulate GA-mediated responses. (A), Germination of the *brm-1* mutant on 10 µM PAC is rescued by the *triple della* mutation. The progeny of *brm-1/BRM* plants were analyzed 10 days after sowing. (B), Phenotypes of 3-week-old plants grown on 2.5 µM PAC. The *brm-1/3xdella* line shows an intermediate growth phenotype. Bar = 5 mm. (C), RT-qPCR analysis of relative transcript levels of the *OFP16, EXP5, CYS2* and *LTP2* genes in 18-d-old wild type, *brm-1*, *ga1-3*, *ga1-3/brm-1*, *ga1-3/3xdella* and *ga1-3/brm-1/3xdella* lines. Transcript levels in the wild type were set to 1. Data are the means ± s.d. of 3 biological replicates. (D), Model of the role of BRM in regulating the expression of GA-responsive genes. BRM positively regulates the *GA3ox1* and *SCL3* genes involved in GA biosynthesis and signaling, and probably through this influences the expression of many GA-responsive genes in the opposite manner to DELLA repressors. In addition, BRM seems to act on a subset of GA-responsive genes independently of DELLA repressors. Also in this case, the effect exerted by BRM is typically in the opposite direction to that of DELLAs and is observed both for genes up- and down-regulated by the SWI/SNF complex (blue and red lines, respectively).

We reasoned that some of the traits of *brm* mutants are probably due to reduction in the levels of active GAs. Traits such as decreased germination and viability in the presence of PAC and delayed flowering under short-days conditions (Figure 1B, Figure 2C) can be reversed by exogenous GAs, which are known to act predominantly through the destruction of DELLA repressors. On the other hand, and as stated above, some GA-related traits of *brm-1* plants, like short roots and reduced plant size, showed additive changes in the double *ga1-3/brm-1* mutant and were only marginally ameliorated by GA treatment of *brm-1* (Figure 2A, Table S1). To further investigate the role of BRM in GA-mediated responses, we constructed a *brm-1/3xdella* line (*brm-1/rga-28/rgl1-2/rgl2-13*), in which three of the five Arabidopsis *DELLA* genes were mutated. Consistent with the observed effects of GAs (Figure 1B), these mutations fully restored the ability of *brm-1/3xdella* mutant plants to germinate on PAC-containing medium (Figure 6A). However, the *triple della* mutation had a less prominent effect on the root length of the *brm-1* plants grown in the presence of PAC (Figure 6B), which is probably due to *brm-1*-specific developmental defects. This interpretation is supported by the observation that the root phenotype is stronger in *brm-1* than in *ga1-3*, and that this phenotype is additive in the *ga1-3/brm-1* double mutant (Figure 4A and C). Similarly small effects on root growth were observed on crossing the *brm-1/ga1-3* mutant with the *3xdella* mutant line (Figure S6A), and upon treatment of *brm-1* or *ga1-3/brm-1* plants with exogenous GAs (Figure 2A and Figure S6B). The strongly repressed growth of *brm-1* on PAC was also attenuated by the *triple della* mutation, and the *brm-1/3xdella* mutant line showed an intermediate phenotype compared with the parental lines, especially later in development (Figure 6B and Figure S6C). While the partial rescue in *brm-1/3xdella* compared with *3xdella* may be caused by higher levels or activity of the remaining two DELLA proteins (GAI and RGL3), it is also possible that BRM exerts some additional DELLA-independent regulatory effect on GA-responsive genes.

### BRM Acts Through Distinct Mechanisms to Regulate GA-responsive Genes

To further investigate the mechanisms by which BRM can regulate GA-responsive genes, we focused on putative GA- and BRM-dependent genes showing enhanced expression in the *ga1-3/brm-1* double mutant. As genes that are responsive to GA are oppositely regulated by gibberellins and DELLAs [58], we examined the effect of the *della* mutations on the expression of selected genes by using *brm-1/3xdella* mutants in the *ga1-3* background. In agreement with the phenotype of *ga1-3*/*brm-1* and the microarray data, the effects of the *ga1-3* and *brm-1* mutations were apparently additive for the genes *EXP5* (AT3G29030) and OFP16 (AT2G32100), and synergistic for *CYS2* (AT2G31980) and *LTP2* (AT2G38530) (Figure 6C). All of these genes were DELLA-responsive, since removal of *RGA*, *RGL1*, and *RGL2* in the *ga1-3* background changed their transcript levels in the wild type direction, although in the case of *EXP5* and *LTP2* they did not reach the wild type level of gene expression (possibly because these genes are also under the control of the GAI and/or RGL3 DELLA proteins). In agreement with the microarray data (Figure 5C), this analysis demonstrated that BRM and DELLAs have opposing effects on the expression of GA-dependent genes. Notably, in the *ga1-3*/*brm-1*/*3xdella* line the expression levels of *EXP5, OFP16, CYS2* and *LTP2* were intermediate between those in the *ga1-3*/*brm-1* and *ga1-3*/*3xdella* mutants, indicating an additive effect of BRM and DELLAs, and suggesting that DELLAs and SWI/SNF can independently regulate the expression of these gene targets.

## Discussion

### BRM Positively Regulates GA Biosynthesis

In this study, we examined the functional links between BRM ATPase, a catalytic subunit required for SWI/SNF-dependent chromatin remodeling, and GA-signaling in Arabidopsis. Our interest in the cross-talk between these two pathways followed the observation that plants with mutations in BRM resemble in several respects mutants with suppressed GA signaling or biosynthesis. Moreover, *brm* mutants showed increased sensitivity to GA biosynthesis inhibition which could be reversed by treatment with exogenous GAs or by mutation of genes encoding the DELLA repressors. Similarly, the delayed flowering of the *brm-1* mutant under short-day conditions reverted to the wild type pattern upon treatment with exogenous GAs. We also found a highly significant overlap between the transcriptional profiles of *brm-1* and GA biosynthesis mutant *ga1-3*. In agreement with these observations, the level of bioactive GA_4_ was considerably reduced in a *brm* null mutant compared with the wild type**,** although it was still more than 3 times higher than in the *ga1-3* mutant. GA_4_ depletion in BRM*-*deficient plants was consistent with the results of qRT-PCR analysis showing that expression of the *GA3ox1* gene, encoding GA 3-oxidase, responsible for the last step of synthesis of bioactive GA_4_, was down-regulated by *brm* mutations (Figure 3A). Moreover, ChIP experiments demonstrated that the promoter of *GA3ox1* is bound by BRM, suggesting that SWI/SNF remodeling is directly involved in transcription of this gene. Part of the complex phenotype of *brm-1* and the large number of GA-related genes found to be misregulated in this mutant can therefore be accounted for by mild GA-deficiency caused mostly by *GA3ox1* downregulation. In agreement with this interpretation, the phenotype of *brm* plants is more similar to that of the semidwarf *ga3ox1* mutant than the severe *ga1-3* mutant [20]. We also hypothesized that the altered expression of other genes involved in GA biosynthesis and signaling in *brm* plants is a consequence of feedback control in response to decreased *GA3ox1* expression and GA content. This was supported by the fact that we were unable to detect BRM on promoters of *GA20ox2* and *GID1b* by ChIP analysis. Moreover, there was a decrease of about 2-fold in the levels of the metabolites GA_12_ and GA_34_ in *brm* (Table 1), similar to that previously described in the *ga3ox1* mutant [20], [62]. The reduction in GA_12_ observed in these studies was explained by increased activity of GA20ox enzymes due to feedback regulation [20], [62]. On the other hand, the increase in the level of the metabolite GA_9_ in *brm* was considerably lower than that reported for *ga3ox1* [20], [62], raising the possibility that BRM can also influence GA biosynthesis by different means.

### BRM Affects GA Signaling

A *ga1-3/brm-1* mutant showed additive or synergistic phenotypes (Figure 4A–E). In agreement with these effects, transcriptional analysis of this double mutant revealed additive or synergistic changes in the expression of many genes that are affected in both *ga1-3* and *brm-1*. Thus, BRM, in addition to promoting GA synthesis, may also counteract the negative effects of DELLAs in a different way. Indeed, we showed that as well as promoting *GA3ox1* expression, BRM also positively and directly regulates the *SCL3* gene, encoding a positive regulator of GA responses that was proposed to act by attenuating DELLA repressors [42], [43]. This suggests that BRM not only regulates GA biosynthesis, but also affects GA signaling. The down-regulation of *SCL3* (Figure 4F) could, at least in part, be responsible for the observed additive effects in the *ga1-3/brm-1* double mutant (at both the phenotypic and transcriptional levels), since the *scl3* null mutation was previously shown to enhance the *ga1-3* phenotype [42], [43].

The presence of BRM on the promoters of *SCL3* and *GA3ox1*, two genes homeostatically regulated by GA signaling components [17], [22], [25], suggests that BRM-containing SWI/SNF complexes might be recruited specifically to these target sequences by transcriptional regulators acting in the GA signaling pathway. Interestingly, a few examples of DNA-binding regulators that are likely to act in GA homeostasis and could potentially serve to recruit chromatin remodeling complexes have been reported [17], [22].

### BRM can Regulate GA-responsive Genes in a DELLA-independent Manner

Our physiological analyses revealed that some GA-related traits of the complex *brm-1* phenotype were not fully reversed by GA treatment nor by a *triple della* mutation (Figure 2A, 6B, and Figure S6). We therefore investigated whether BRM could act on GA-responsive genes independently of DELLAs. By comparing the transcriptional response of putative GA- and BRM-dependent genes in *ga1-3/brm-1* and *ga1-3/brm-1/3xdella* mutants, we identified genes that were affected in an additive manner by BRM and DELLAs, suggesting that BRM can also control GA-responsive genes by acting in parallel to DELLA repressors (Figure 6D). Alternatively, the partial rescue in *brm-1/3xdella* compared with *3xdella* may be caused by higher levels or activity of the remaining two DELLA proteins, GAI and RGL3. It should also be noted that it is not yet known whether *SCL3* down-regulation could also influence *brm-1/3xdella* phenotypes. While the *rga* mutation was shown to be epistatic to the *scl3* null mutation in root length assays in the presence of PAC, this epistasis was found to be only partial at later developmental stages [42], [43]. Clearly, further studies – like ectopic expression of *SCL3* in the *brm* mutant background – are required to determine the extent to which the decreased *SCL3* level accounts for the GA-insensitive part of the *brm* phenotype. Finally, while the positive regulatory function of BRM in respect to *GA3ox1* and *SCL3* is fully consistent with the observed opposite effects of BRM and DELLAs on GA-responsive genes, it is still surprising, given the occurrence of GA-independent regulation by BRM, that the expression of over 90% of the overlapping genes behaves similarly (up- or down-regulation with respect to the wild type) in each of the single mutants (*ga1-3* and *brm-1*) and in the double *ga1-3/brm-1* mutant (Figure 5C). This may indicate that there are other, as yet unrevealed, levels of functional interaction between BRM and GA signaling.

### BRM Affects GA-mediated Responses in Diverse Ways

Taken together, the findings of this study implicate BRM as a positive regulator of GA-mediated responses and reveal diverse (both direct and indirect) interactions between SWI/SNF BRM ATPase and the GA pathway. This resembles the complex involvement of SWI/SNF chromatin remodeling in the regulation of flowering [13], where BRM appears to repress important flowering regulators that act in different genetic pathways. By highlighting the interactions between BRM and the GA pathway, our results disclose another layer of complexity and suggest a role for BRM-dependent SWI/SNF chromatin remodeling in the integration of GA-controlled responses with other signaling pathways. A candidate gene for such regulation is *GA3ox1*, shown in this study to be a direct target of BRM, and whose transcription is tightly regulated by both developmental and environmental stimuli [17], [19], [22].

Interestingly, mutants in another chromatin remodeling factor PICKLE (PKL), a chromodomain-containing Snf2-type ATPase, are also semi-dwarf, resembling GA-response mutants, and their characteristic pickle-root phenotype is greatly enhanced by treatment with GA-biosynthesis inhibitors and decreased by GA treatment [63]. PKL has been shown to control a large number of GA-responsive genes by acting in parallel to GA signaling [64]. However, in contrast to the situation in the *brm* mutant, *pkl* plants were found to have increased levels of active gibberellin. Thus, both of these chromatin remodeling complexes seem to act as positive regulators of the GA pathway, although probably by different mechanisms.

## Materials and Methods

### Plant Lines and Growth Conditions

*Arabidopsis thaliana* wild type and all mutant lines were of the Columbia-0 (Col-0) ecotype. The *brm-1*, *brm*-*3,* and *brm-6* mutant alleles were characterized previously [9], [46], [44]. The *ga1-3* line introgressed into Col-0 and the *ga1-3/rga-28/rgl1-2/rgl2-13* line [27] were kindly provided by Dr. Tai-ping Sun. To obtain *ga1-3/brm-1* and *ga1-3/brm-1/rga-28/rgl1-2/rgl2-13* (*ga1-3/brm-1/3xdella*) lines, heterozygous *brm-1* mutant plants were crossed with *ga1-3* and *ga1-3/rga-28/rgl1-2/rgl2-13* homozygous lines, respectively, followed by PCR screening of mutant alleles in the segregating populations. The *triple della* (*rga-28/rgl1-2/rgl2-13)* and *brm-1/3xdella* lines were obtained by screening the same population. Primers used for genotyping are listed in Table S8. Due to the sterility of all lines containing the homozygous *brm-1* mutation, segregating progeny were sown for each analysis and genotyped. For analysis of plants with the *ga1-3* background, seeds were imbibed in 100 µM GA_3_ for 3 d at 4°C and then washed thoroughly in water before sowing. Plants were grown under long-day (LD; 16 h light/8 h dark) or short-day (SD; 8 h light/16 h dark) conditions at 18–23°C, with 70% humidity and 200 µM m^−2^ s^−1^ light intensity. Seedlings were cultivated in medium containing ½ Murashige and Skoog (MS) salts (Sigma-Aldrich), 0.5% (w/v) sucrose and 0.8% (w/v) agar, pH 5.8, or in soil.

### PAC and GA Treatment

For germination assays, seeds of the wild type and *brm-3*, *brm-1*/*BRM*, *brm-1/BRM/3xdella* and *3xdella* genotypes were sown on MS plates containing different concentrations of PAC or 10 µM PAC +10 µM GA_3_. Segregating progeny of *brm-1*/*BRM* plants were genotyped using primers specific for the wild type and mutant alleles in order to confirm or exclude the *brm-1* genetic background. To analyze growth responses of *brm-1* or *brm-1/3xdella* mutants to PAC, seeds of wild type, *brm-1*/*BRM*, *brm-1/BRM/3xdella* and *3xdella* genotypes were sown on MS plates containing 10 µM PAC or 10 µM PAC +10 µM GA_3_ and cultivated for 25 days. In order to promote equal germination of all seeds, they were pre-incubated with 100 µM GA_3_, then rinsed thoroughly and sown on PAC-containing media. To analyze the GA response, plants were grown in soil and treated with 10 µM GA_3_ by spraying twice a week, or they were grown on plates containing ½ MS medium supplemented with 1 µM or 2 µM GA_4_, placed vertically under long-day conditions at 22°C. For the set of data for which differences were small, statistical significance was estimated by determining P value using Student’s t-test.

### Quantification of Plant Hormones

Aerial parts of soil-grown wild type, *brm-6* and *ga1-3* plants were harvested at the end of the day and immediately frozen in liquid nitrogen. Plant hormones were quantified according to Plackett et al., (2012) [51] by using a 6410 Triple Quad LCMS (Agilent Technologies, Santa Clara, CA, USA) with an Agilent 1200 series rapid resolution liquid chromatography system fitted with a ZORBAX Eclipse XDB-C18 column (1.8 µm, 2.1×50 mm).

### Microarray Transcriptome Analysis

Total RNA was extracted from shoots of 18-d-old wild type, *brm-1, ga1-3,* and *ga1-3/brm-1* seedlings using the RNeasy plant mini kit (Qiagen) according to the manufacturer’s protocol, followed by TURBO DNase treatment (Ambion). The quantity and quality of the isolated RNA was determined using a NanoDrop ND1000 spectrophotometer (Nanodrop technologies) and RNA integrity was assessed with a Bioanalyzer 2100 (Agilent Technologies). 100 ng of RNA were used for aRNA synthesis with a GeneChip 3′ IVT - Express Kit (Affymetrix), and 15 µg of labeled and fragmented aRNA were hybridized with Arabidopsis ATH1 genome arrays, according to the manufacturer’s recommendations (Affymetrix). Three biological replicates were examined for each genotype.

### Microarray Data Analysis

Microarray hybridization signals were recorded and processed using *Affymetrix*® *GeneChip*® *Command Console*® Software (AGCC). All processed samples passed the quality control tests. The resulting CEL files were further analyzed with the Partek Genomics Suite (Partek). A GC-RMA normalization was conducted. Principle Components Analysis (PCA) for all genes revealed significant separation based on genotype. To exclude genes that were not expressed in the plant material, a non-specific filter was applied using the MAS5.0 algorithm. Only those genes identified as “present” in at least one of the three replicates of a genotype were included in further analysis, and 16,824 of 22,810 passed the filtering criteria. A two-way ANOVA was performed and genes with a false discovery rate (FDR) of <0.05 were considered significantly altered in their expression in the mutants compared with the wild type. The gene lists were then filtered to select those with a fold change of >1.5. Gene list comparisons were performed using the Partek Genomics Suite. Gene expression data shown as heat-maps was standardized by the default method in the software (z-score conversion) to receive values between −2 and +2. Overlap analysis of genes differentially expressed in the *brm-1* and *ga1-3* mutants was performed using Fisher’s exact test. Gene ontology analyses were performed with the FatiGO [65] (Table 2) and GOrilla [66] (Figure S5) tools. The microarray data have been deposited in Gene Expression Omnibus and are accessible through GEO Series accession number GSE26848.

### Real-Time RT-qPCR Analyses

Aerial parts of 18-d-old seedlings of wild type, *brm-1*, *ga1-3*, *ga1-3/brm-1*, *ga1-3/rga/rgl1/rgl2* and *ga1-3/brm-1/rga/rgl1/rgl2* lines were used for analyses. RNA was extracted from plant material using the RNeasy plant mini kit (Qiagen) and DNA was removed by TURBO DNase-treatment (Ambion). A first-strand cDNA synthesis kit (Roche) was used to prepare cDNA from 1 µg of RNA. Aliquots (2 µl) of 5-fold diluted cDNA samples were used as templates in 20 µl reactions containing LightCycler 480 SYBR Green I Master mix (Roche) and specific primers for PCR amplification in a LightCycler 480 System (Roche), as recommended by the manufacturer. The final primer concentrations were 0.5 µM and the annealing temperature was set at 58–60°C. The RT-qPCR data were analyzed with LightCycler 480 Software version 1.3. *PP2A* and *GAPc* housekeeping genes were used as normalization controls and gave similar results. Each experiment was performed using at least two independent biological replicates, and the specificity of real-time PCR products was confirmed by melting curve analysis and agarose gel electrophoresis. For the set of data for which differences were small (Figure 3A), statistical significance was estimated by determining P value using Student’s t-test. Specific primers used in qPCR reactions are listed in Table S8.

### Chromatin Immunoprecipitation

ChIP experiments were performed as described by Gendrel et al. (2005) [67] with some modifications. Aerial parts of 15-d-old seedlings of the wild type and *brm-1* mutant (negative control) were used as the source of chromatin. Anti-BRM antibody [44] was bound to Dynabeads Protein A (Invitrogen) and incubated with aliquots of 10-fold diluted chromatin (∼100 µg of DNA). The isolated DNA was resuspended in 100 µl of water. ChIP enrichment of putative BRM targets was determined by qPCR using LightCycler 480 SYBR Green I Master mix (Roche). Reactions were performed with 2 µl of immunoprecipitated DNA as template. A standard curve was established for each pair of primers. The amount of ChIP DNA was calculated based on the standard curve and then normalized to the PP2A promoter sequence signal (control locus) for each sample. Fold enrichment of each region in the wild type was adjusted relative to the *brm-1* sample. In independent BRM-ChIP experiments, enrichments were also determined by subjecting the input and immunoprecipitated DNA to PCR and visualizing amplified bands by ethidium bromide staining after separation on agarose gels (Fig. S3). The *2S2-u* sequence was used as a positive control as it has been shown to bind BRM protein [12]. 18S rDNA served as negative control for BRM binding. Primers used in ChIP experiments are listed in Table S8.

## Supporting Information

Figure S1 **Examples of phenotypic traits of the** ***brm-1*** **null mutant resembling those of mutants with suppressed GA biosynthesis or signaling.** (A, B), Semi-dwarfism and dark green coloration. (C), Short and branched roots. (D), Closed flowers. (E), Underdeveloped stamens. (F), *brm-1* homozygous mutants germinate and are viable when grown on 10 µM PAC-containing medium supplemented with 10 µM GA_3_. At this concentration of GA_3_, the growth phenotype of wild type plants did not fully recover. 20-d-old plants are shown. Bar = 5 mm.
(TIF)

Figure S2 **Comparison of** ***brm-3*** **and** ***brm-1*** **mutants.** 14- and 20-d-old plants grown on MS medium (A) or in soil (B) under LD conditions are shown, respectively. Bars = 5 mm.
(TIF)

Figure S3 **ChIP analysis of potential BRM targets and control genes.** The *2S2-u* promoter [12] and 18S rDNA served as positive and negative controls for BRM binding, respectively. Primer sequences used in ChIP analysis are listed in Table S8.
(TIF)

Figure S4 **Flowering of the** ***ga1-3/brm-1*** **double mutant.** The *ga1-3/brm-1* mutant is usually unable to flower under long-day conditions (A, B). Treatment with 10 µM GA_3_ restores its ability to flower (C). 36-d-old (A) and 54-d-old (B, C) plants are shown.
(TIF)

Figure S5 **Functional analysis of genes commonly regulated by BRM and GAs**. (A, B), Genes misregulated in *ga1-3* and *brm-1* mutants, classified based on Gene Ontology (GO) categories of biological processes (A) and molecular function (B). Charts were generated using the Gene Ontology Enrichment Analysis and Visualization tool (http://cbl-gorilla.cs.technion.ac.il) [65]. There were only 18 genes with the “gibberellin-responsive” GO term in the *ga1-3* microarray dataset; 8 of them were also present in the overlapping gene-set. (C), Expression levels of genes encoding carboxylesterases and pectin-related enzymes in microarray data for *ga1-3*, *brm-1* and *ga1-3/brm-1* lines.
(TIF)

Figure S6 **Effect of DELLA mutations or GA treatment on** ***brm-1*** **and** ***ga1-3/brm-1*** **mutant phenotypes.** (A), Root length of *ga1-3/brm-1/3xdella* compared with *ga1-3/3xdella* plants. 12-d-old plants are shown. (B), Root length of 8-d-old *ga1-3/brm-1* plants compared with *ga1-3* plants grown in the presence of 2 µM GA_4_. (C), Growth phenotype of *brm-1/3xdella* plants grown on 10 µM PAC. 40-d-old plants are shown. Bars = 5 mm.
(TIF)

Table S1 **Effect of GA application on size and flowering of** ***brm-1*** **plants.**
(DOCX)

Table S2 **Genes that exhibit significantly different expression in** ***brm-1*** **mutant comparing to wild type.**
(XLSX)

Table S3 **Genes that exhibit significantly different expression in** ***ga1-3*** **mutant comparing to wild type.**
(XLSX)

Table S4 **Genes that exhibit significantly different expression in** ***ga1-3/brm-1*** **mutant comparing to wild type.**
(XLSX)

Table S5 **Genes showing differential expression both in** ***brm-1*** **and** ***ga1-3*** **mutants.**
(XLSX)

Table S6 **Differentially expressed genes in** ***ga1-3/brm-1*** **mutant that are also changed in the same direction in** ***brm-1*** **and** ***ga1-3*** **.**
(XLSX)

Table S7 **Genes from overlapping list showing enhanced misexpression in** ***ga1-3/brm-1*** **comparing to** ***brm-1*** **and** ***ga1-3*** **.**
(XLSX)

Table S8 **Oligonucleotides used in genotyping, RT-qPCR and ChIP.** [**68]**–[70] **.**
(DOCX)
