# Supplementary figures and images for: BRAHMA ATPase of the SWI/SNF Chromatin Remodeling Complex Acts as a Positive Regulator of Gibberellin-Mediated Responses in Arabidopsis
Source: PLoS One. 2013 Mar 11;8(3):e58588. doi: 10.1371/journal.pone.0058588 (PMC3594165; doi:10.1371/journal.pone.0058588)

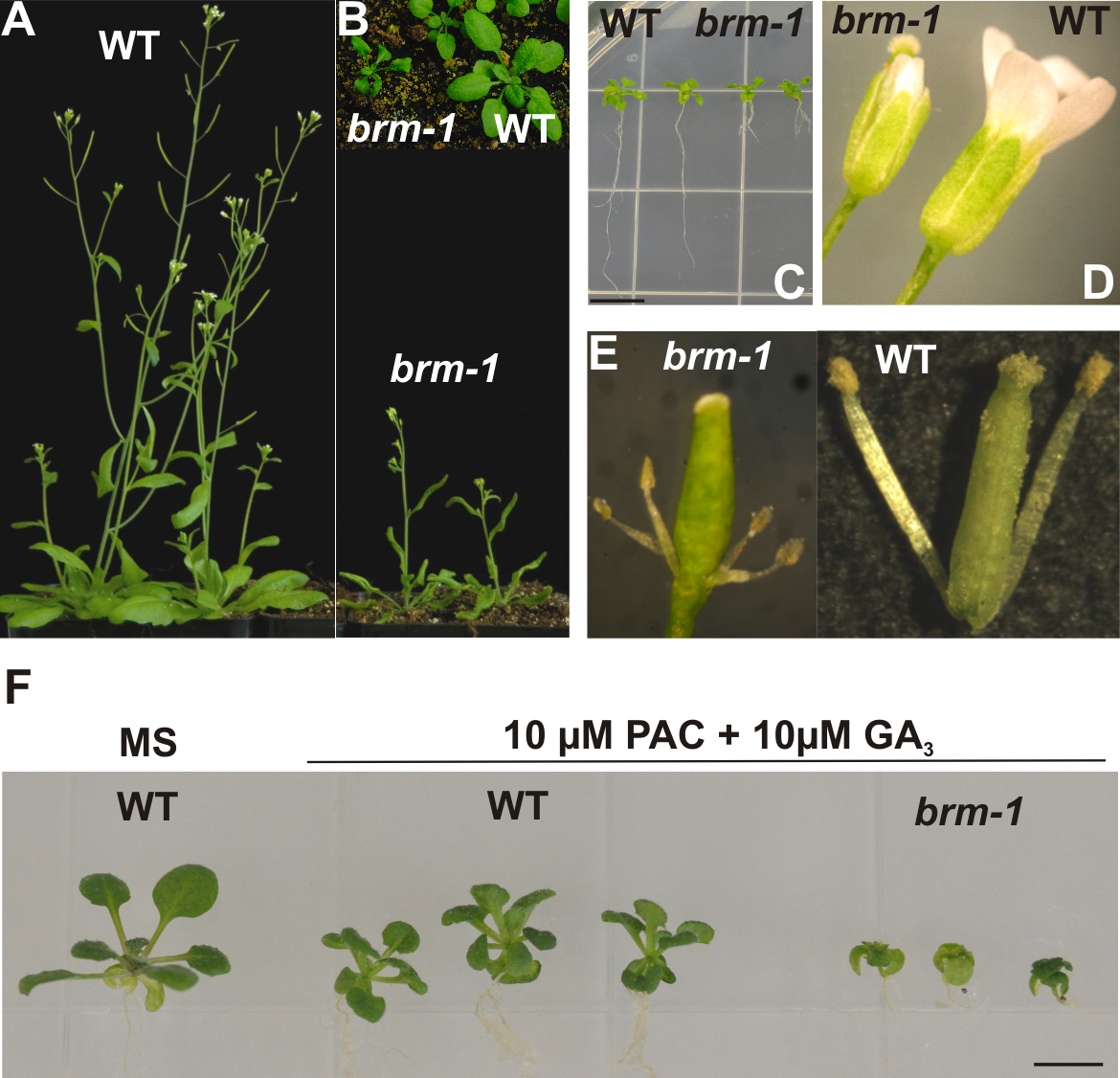

Supplement: Figure S1 — Examples of phenotypic traits of the brm-1 null mutant resembling those of mutants with suppressed GA biosynthesis or signaling. (A, B), Semi-dwarfism and dark green coloration. (C), Short and branched roots. (D), Closed flowers. (E), Underdeveloped stamens. (F), brm-1 homozygous mutants germinate and are viable when grown on 10 µM PAC-containing medium supplemented with 10 µM GA3. At this concentration of GA3, the growth phenotype of wild type plants did not fully recover. 20-d-old plants are shown. Bar = 5 mm. (TIF) [file pone.0058588.s001.tif]

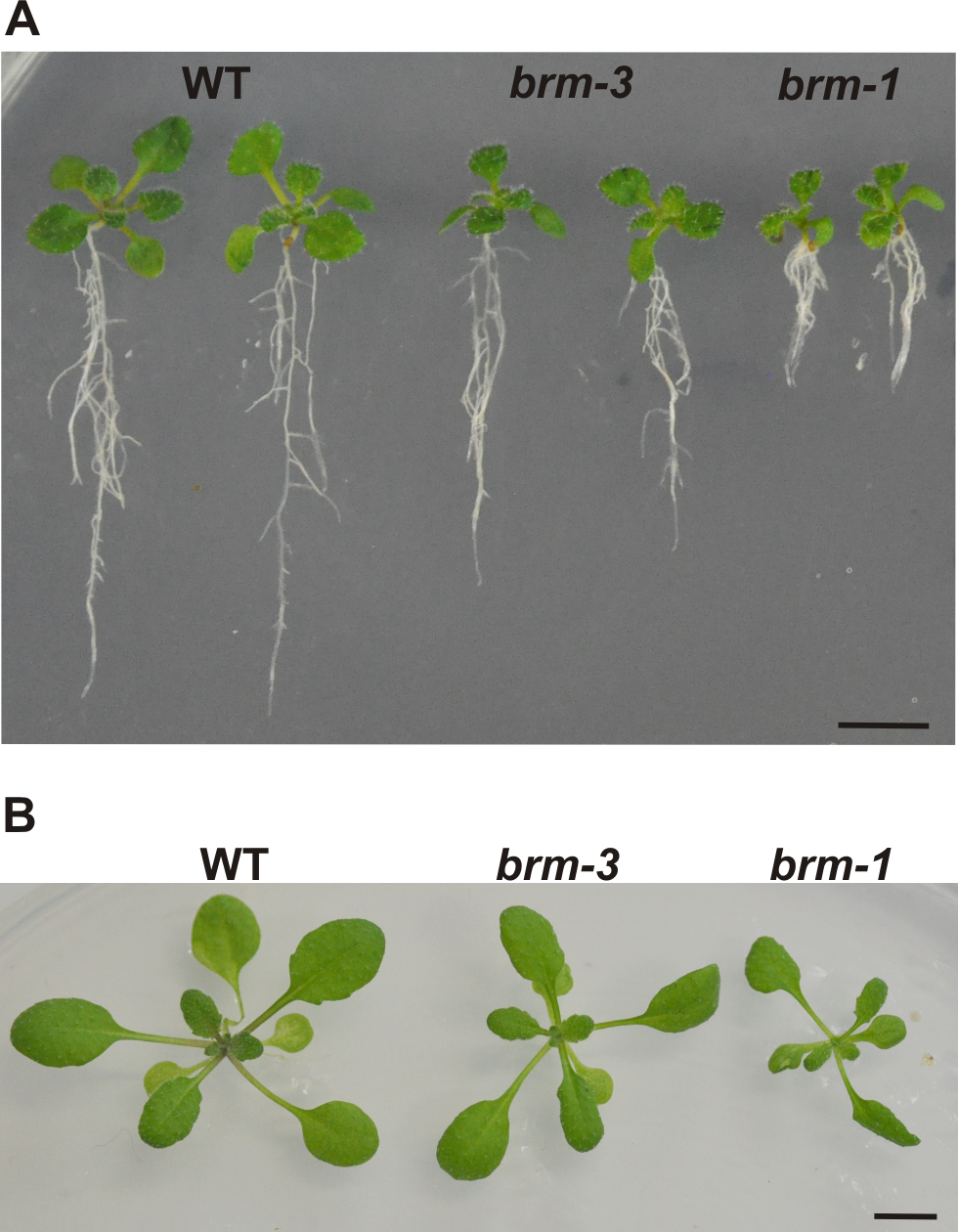

Supplement: Figure S2 — Comparison of brm-3 and brm-1 mutants. 14- and 20-d-old plants grown on MS medium (A) or in soil (B) under LD conditions are shown, respectively. Bars = 5 mm. (TIF) [file pone.0058588.s002.tif]

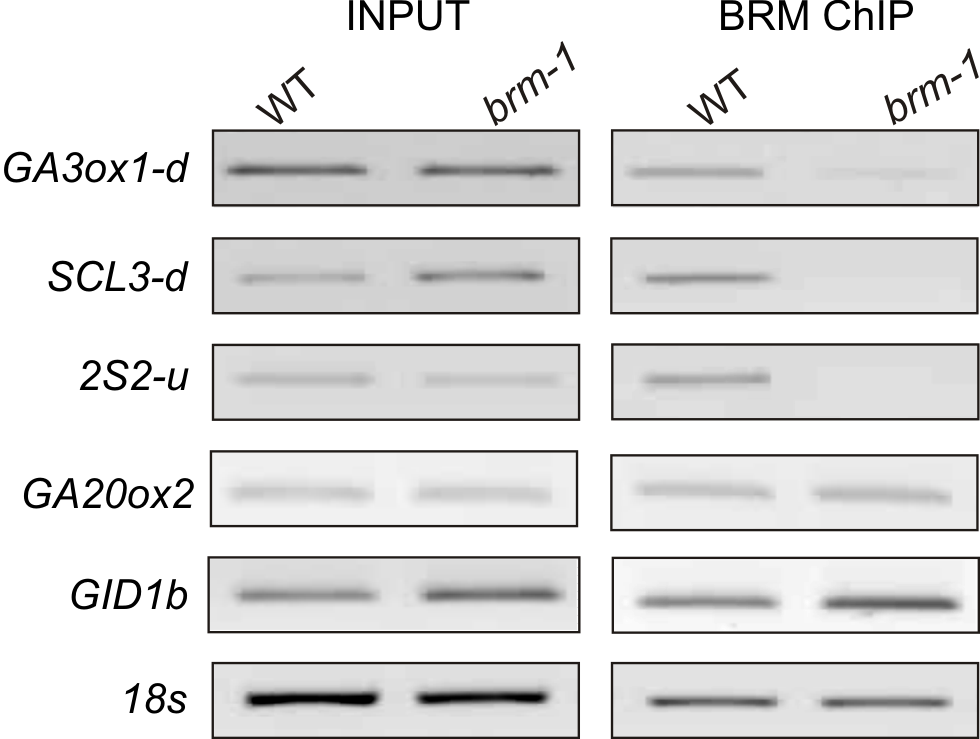

Supplement: Figure S3 — ChIP analysis of potential BRM targets and control genes. The 2S2-u promoter [12] and 18S rDNA served as positive and negative controls for BRM binding, respectively. Primer sequences used in ChIP analysis are listed in Table S8. (TIF) [file pone.0058588.s003.tif]

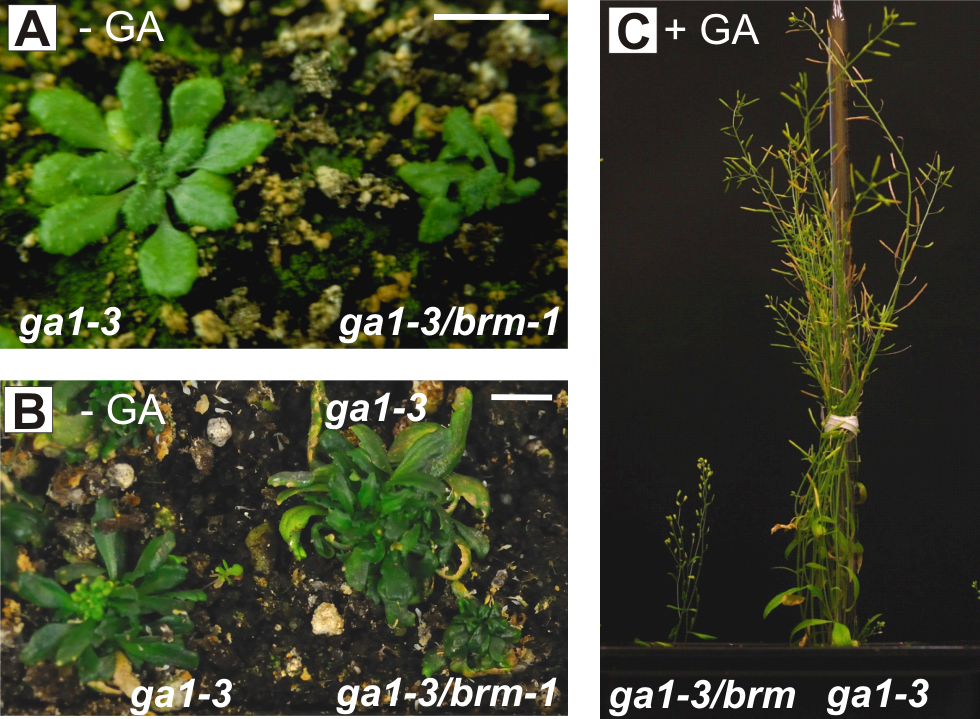

Supplement: Figure S4 — Flowering of the ga1-3/brm-1 double mutant. The ga1-3/brm-1 mutant is usually unable to flower under long-day conditions (A, B). Treatment with 10 µM GA3 restores its ability to flower (C). 36-d-old (A) and 54-d-old (B, C) plants are shown. (TIF) [file pone.0058588.s004.tif]

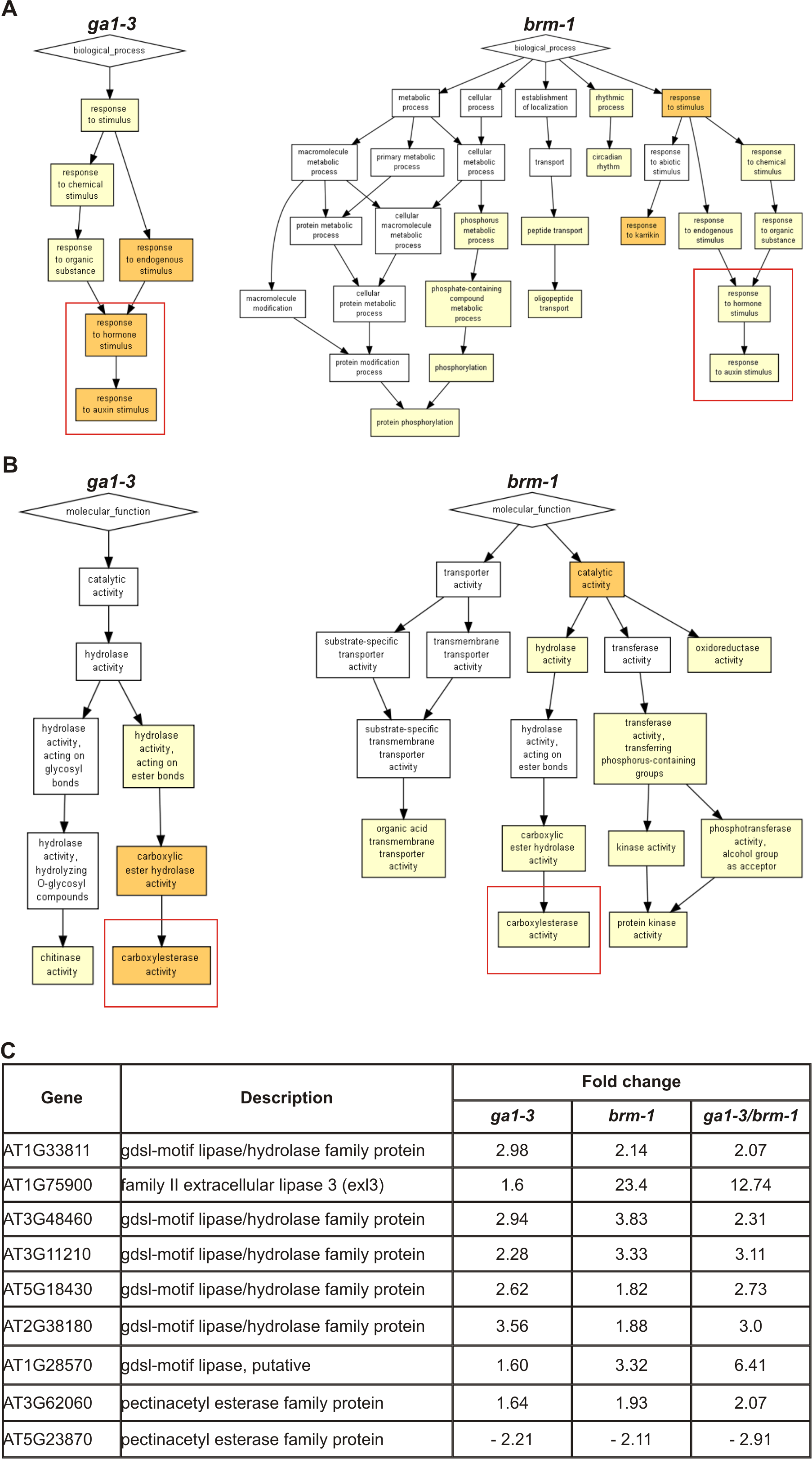

Supplement: Figure S5 — Functional analysis of genes commonly regulated by BRM and GAs. (A, B), Genes misregulated in ga1-3 and brm-1 mutants, classified based on Gene Ontology (GO) categories of biological processes (A) and molecular function (B). Charts were generated using the Gene Ontology Enrichment Analysis and Visualization tool (http://cbl-gorilla.cs.technion.ac.il) [65]. There were only 18 genes with the “gibberellin-responsive” GO term in the ga1-3 microarray dataset; 8 of them were also present in the overlapping gene-set. (C), Expression levels of genes encoding carboxylesterases and pectin-related enzymes in microarray data for ga1-3, brm-1 and ga1-3/brm-1 lines. (TIF) [file pone.0058588.s005.tif]

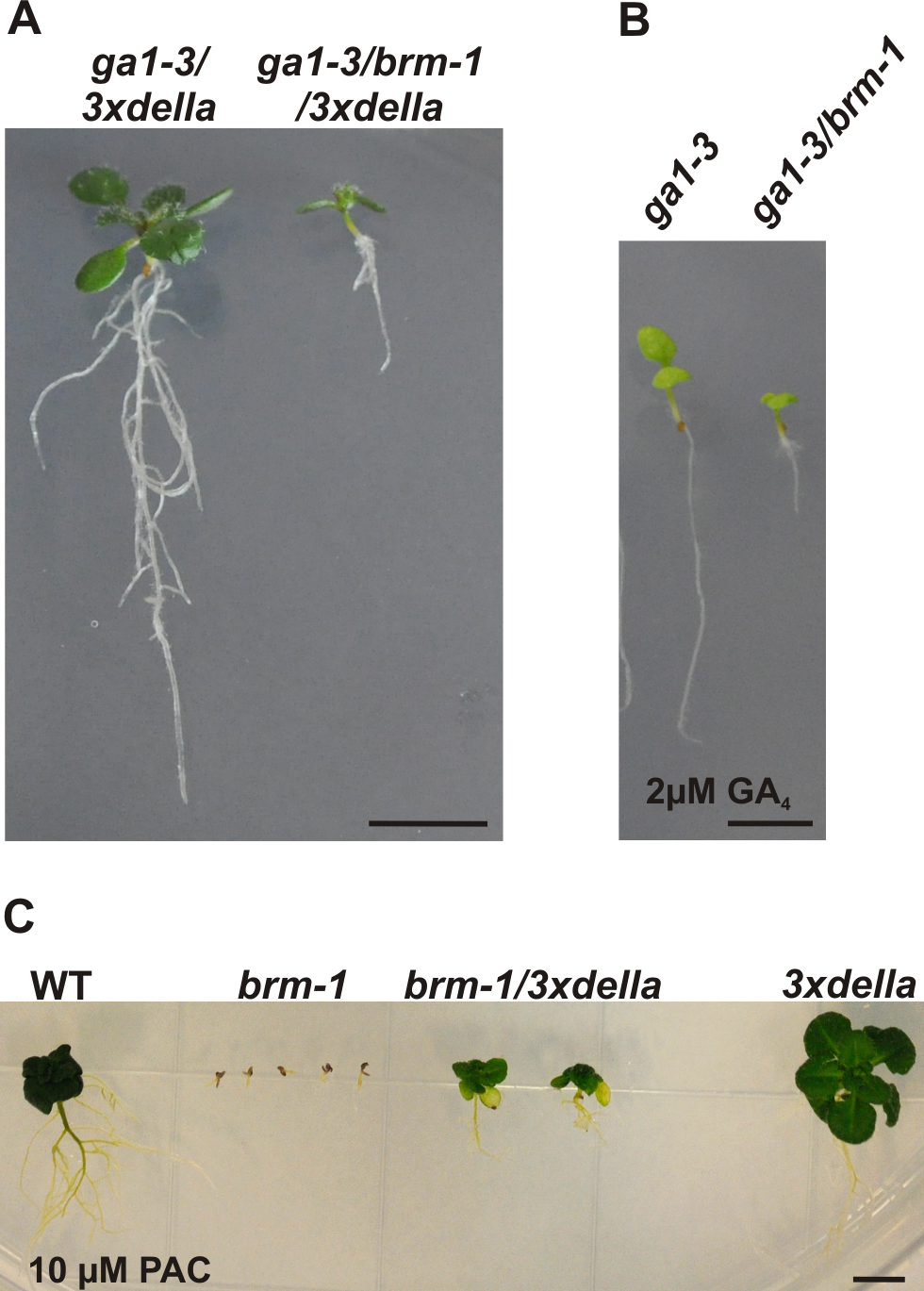

Supplement: Figure S6 — Effect of DELLA mutations or GA treatment on brm-1 and ga1-3/brm-1 mutant phenotypes. (A), Root length of ga1-3/brm-1/3xdella compared with ga1-3/3xdella plants. 12-d-old plants are shown. (B), Root length of 8-d-old ga1-3/brm-1 plants compared with ga1-3 plants grown in the presence of 2 µM GA4. (C), Growth phenotype of brm-1/3xdella plants grown on 10 µM PAC. 40-d-old plants are shown. Bars = 5 mm. (TIF) [file pone.0058588.s006.tif]
