# Supplementary material for: BRAHMA ATPase of the SWI/SNF Chromatin Remodeling Complex Acts as a Positive Regulator of Gibberellin-Mediated Responses in Arabidopsis
Source: PLoS One. 2013 Mar 11;8(3):e58588. doi: 10.1371/journal.pone.0058588 (PMC3594165; doi:10.1371/journal.pone.0058588)
Supplement: Table S1 — Effect of GA application on size and flowering of brm-1 plants. (DOCX) [file pone.0058588.s007.docx]

**Table S1. Effect of GA application on size and flowering of *brm-1* plants.**

|  | wild type  - GA | wild type  + GA | *brm-1*  - GA | *brm-1*  + GA |
| --- | --- | --- | --- | --- |
| Rosette radius at maturity (LD) [cm] | 4.3 ± 1.2 | 4.9 ± 0.7 | 3.1 ± 0.5 | 3.3 ± 0.4 |
| Height at maturity (LD) [cm] | 31.9 ± 6.8 | 37.2 ± 5.4 | 18.1 ± 3.5 | 25.1 ± 4.0 |
| Leaf no. at flowering (LD) | 11.6 ± 1.2 | 9.8 ± 0.8 | 9.9 ± 1.1 | 9.4 ± 0.8 |
| Days to flowering (LD) | 19.6 ± 2.6 | 19.3 ± 2.4 | 26.7 ± 3.3 | 25.2 ± 2.7 |
| Leaf no. at flowering (SD) | 54.2 ± 6.1 | 32.8 ± 2.7 | 22.8 ± 2.7 | 18.8 ± 1.6 |
| Days to flowering (SD) | 60.3 ± 4.1 | 42.6 ± 2.4 | 71.1 ± 5.8^a^ | 44.2 ± 2.9 |

Plants were grown in soil and treated with 10 μM GA_3_ by spraying twice a week. Data are means ± s.d. At least 15 plants of each line/condition were scored. LD – long-day conditions. SD – short-day conditions. ^a^ about 20% of *brm-1* plants grown under SD conditions never flowered, consistent with the data of Farrona et al. (2011) [13]. These plants were not included in calculations.
