# Supplementary material for: BRAHMA ATPase of the SWI/SNF Chromatin Remodeling Complex Acts as a Positive Regulator of Gibberellin-Mediated Responses in Arabidopsis
Source: PLoS One. 2013 Mar 11;8(3):e58588. doi: 10.1371/journal.pone.0058588 (PMC3594165; doi:10.1371/journal.pone.0058588)
Supplement: Table S8 — Oligonucleotides used in genotyping, RT-qPCR and ChIP. [68] –[70] . (DOCX) [file pone.0058588.s014.docx]

**Table S8.** **Primers used in genotyping, RT-qPCR, and ChIP.**

| **Primers used for genotyping** | | | |
| --- | --- | --- | --- |
| **Primer pair** | **Sequences (5’-3’)** | **Method** | **Note** |
| Mut1L  Mut1R | GATTTCCCCAAATTCGATGC  ATCACCCACGGCGCCTATTCCTAAT | Genotyping *BRM* WT allele | Primers described in [44] |
| Lba1  Mut1L | TGGTTCACGTAGTGGGCCATCG  above | Genotyping *brm-1* mutant allele | Primers described in [44] |
| ga1-3wtF  ga1-3wtR | TTTGGCCCAACACACAAACAAACCTT  AAGCTTCGAACTCAAGGTTCTA | Genotyping *GA1* WT allele | Primers described in [68] |
| ga1-3mutF  ga1-3mutR | AATGAGATTCATGTTTGGC  TTTCTTCATACCACCTGCGTTC | Genotyping *ga1-3* mutant allele | this work and [68] |
| RGAwtF  RGAwtR | CAGCTAAGCATCCGATTTGC  TCACATAGAGAAGTCACATG | Genotyping *RGA* WT allele | Primers described in [27] |
| RGAwtF  Lba1 | above  above | Genotyping *rga-28* mutant allele | this work and [27] |
| RGL1wtF  RGL1wtR | GGTTCTTGGTGATGGAATCTCGA  TTATTCCACACGATTGATTCGCCA | Genotyping *RGL1* WT allele | Primers described in [27] |
| RGL1wtF  RGL1mutRB | Above  TGATAGTGACCTTAGGCGACTTTTGAACGC | Genotyping *rgl1-2* mutant allele | this work and [27] |
| RGL2wtF  RGL2wtR | TGCGGTACCATGAAGAGAGGATACGGAG  ATCGGTACCGCCGCGACTCAGG | Genotyping *RGL2* WT allele | Primers described in [27] |
| RGL2wtF  Lba1 | Above  Above | Genotyping *rgl2-13* mutant allele | this work and [27] |
| **Primers used for RT-qPCR** | | | |
| qPP2A-F  qPP2A-R | TATCGGATGACGATTCTTCGTGCAG  GCTTGGTCGACTATCGGAATGAGAG | *PP2A* RT-qPCR | Primers described in [44] |
| GAPc-F  GAPc-F | AGCTGCTACCTACGATG  CACACGGGAACTGTAAC | *GAPc* RT-qPCR | Primers described in [27] |
| GA3ox1-F2  GA3ox1-R2 | CCATTCACCTCCCACACTCT  GCCAGTGATGGTGAAACCTT | *GA3ox1* RT-qPCR | Primers described in [20] |
| GA3ox2-qF2  GA3ox2-qR2 | TGGTCCGAAGGTTTCAC  GGGTCGAGTCTGTATGG | *GA3ox2* RT-qPCR | Primers described in [20] |
| GA20ox1-qF2  GA20ox1-qR2 | GATCCATCCTCCACTTTAGA  GTGTATTCATGAGCGTCTGA | *GA20ox1* RT-qPCR | Primers described in [21] |
| GA20ox2-qF2  GA20ox2-qR2 | AAAACCCGGTGAGAGTTGTG  TAAGCCCAGAAGCTCCATGA | *GA20ox2* RT-qPCR | this work |
| GA2ox1-qF2  GA2ox1-qR2 | TGAGGACGAGAGGTTGTACGA  TCCTTTCGAATTGTTGAAGCC | *GA2ox1* RT-qPCR | Primers described in [21] |
| GA2ox2-qF  GA2ox2-qR | GGAACACACAGACCCACAGAT  TGTATCGGCTAAGACCCTGTG | *GA2ox2* RT-qPCR | this work |
| GID1a-qF  GID1a-qR | GTTTGGTGGGAATGAGAGAACG  CTAAACGCCTCACTGTTCTTCC | GID1a RT-qPCR | Primers described in [69] |
| GID1b-qF  GID1b-qR | CATCCAGCATGTAATCCCTTTGG  CACTTGTGGAAACTGTACACAACC | GID1b RT-qPCR | Primers described in [69] |
| GID1c-qF  GID1c-qR | ATGATGATGGATGGGCTGTT  TTCGGTCCCTCCAAACATAG | GID1c RT-qPCR | this work |
| SCL3-qF  SCL3-qR | ATTATGCGATGTTGCAGG  ATTACACCCACACCAGAC | *SCL3* RT-qPCR | Primers described in [58] |
| OFP16-qF2  OFP16-qR2 | TCTTCCTCCACCACAACCACTC  ATTCTCTTGACCGGGGTTCTGG | *OFP16* RT-qPCR | this work |
| EXP5-qF  EXP5-qR | AGAGAAGTGGTGGGATAAGGTTC  AACAAACGACAGACTTTGACCAT | *EXP5* RT-qPCR | this work |
| CYS2-qF  CYS2-qR | AGGTTTTCTGGTGATCGCTGTC  TGGATTCGAAATTGCCGTGTCTG | *CYS2* RT-qPCR | this work |
| LTP2-qF  LTP2-qR | GAACGCGCTTATGAGTTGTG  CTTTAGCGGCAGATTGAAGG | *LTP2* RT-qPCR | this work |
| **Primers used for ChIP** | | | |
| GA3ox1A2-F  GA3ox1A2-R | TGAATTTGGATCATGAAGACG  AGCGCAAGAGGCGTATAGAA | GA3ox1-d ChIP-PCR and ChIP-qPCR | Amplifies region 1490 -1268 upstream from ATG |
| 3ox1 –C-F | gacaacccatcatgtgtatgtatc  CATATGTTAAAGCACTTGTTTTGGT | GA3ox1-p ChIP-qPCR | Amplifies region 1490 -1268 upstream from ATG; primers described in [70] |
| SCL3-900-F2  SCL3-900-R2 | TAATGCCAAATGGGTTCATTG  GCTAATGTTGCATTTTCATTTTC | SCL3-d ChIP-PCR and ChIP-qPCR | Amplifies region 1096-892 upstream from ATG; |
| SCL3-5UTR2-F  SCL3-5UTR2-R | TCTCTGCAAACCCTAGTTCCTC  TGAAGGCCAAAAGCTTGATT | SCL3-p ChIP-qPCR | Amplifies region 194-2 upstream from ATG; |
| PP2A-Prom-F  PP2A-Prom-R | ttccgatattttctgaggaagaag  gggcctgtcaaagataagaactaa | PP2A ChIP-qPCR | Amplifies region 600 -454 upstream from ATG |
| ChIP-2S2u-F  ChIP-2S2u-R | CACATACCACACCTCATCGTG  CCATTTGTCTCTACGCATTTGA | *2S2-u* ChIP PCR | Amplifies target sequence for *BRM* as described previously [12] |
| 18S-F  18S-R | ATACGTGCAACAAACCC  CTACCTCCCCGTGTCA | *18S* rDNA ChIP PCR | Primers described in [58] |
